# Potent and Long‐Lasting Immunogenicity Generated by LNP‐mRNA gE Antigen Against Varicella Zoster Virus via an AI‐Assisted Pipeline

**DOI:** 10.1002/advs.202510913

**Published:** 2025-12-25

**Authors:** Kai Dong, Fang Liu, Dawei Wang, Yanfen Li, Man Zhang, Yuting Zhou, Gengshen Song

**Affiliations:** ^1^ Beijing Youcare Kechuang Pharmaceutical Technology Co., Ltd. Beijing China

**Keywords:** antigen localization, cellular immune responses, glycoprotein E (gE), mRNA vaccine, untranslated regions (UTRs), varicella zoster virus (VZV)

## Abstract

Herpes zoster (HZ), caused by varicella zoster virus (VZV) reactivation commonly in elderly/immuno‐compromised individuals, leads to tremendous social burden. Despite approved vaccines, there is an urgent need for longer‐lasting and more effective HZ vaccines. This study designs a novel mRNA‐LNP vaccine targeting VZV glycoprotein E (gE), with the assistance of AI technology, achieving effective and enduring prevention against VZV infection with favorable safety. From numerous empirically designed antigen sequences, AI‐based screening identifies promising candidate variants, which are systematically evaluated based on their humoral and cellular immune responses in BALB/c mice. The study further explores the underlying correlation between the cellular localization and immunogenicity of the vaccine candidates and screens 5’UTR elements affecting mRNA expression. A 4‐week administration interval and immunogenicity are subsequently validated via detection of gE‐specific IgG antibodies and CD4^+^ T‐cell responses in mice. Notably, the vaccine VZV‐gEmD elicits strong humoral response, including gE‐specific IgG antibodies and VZV‐specific antibodies in rhesus macaques. The CD4^+^ T‐cell responses, critical for VZV reactivation protection, are significantly stronger and longer lasting with VZV‐gEmD than with Shingrix. These findings highlight its potential as an effective prophylactic vaccine for HZ and provide confidence for the utilization of this mRNA platform in VZV vaccine development.

## Graphical abstract

This study designs a novel mRNA‐LNP vaccine targeting VZV glycoprotein E (gE) for herpes zoster via an AI‐assisted pipeline. Validated in mice and rhesus macaques, the mRNA‐LNP vaccine shows strong humoral and cellular immune responses, with CD4^+^ T‐cell responses more effective and durable than Shingrix, offering a promising prophylactic option.

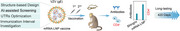

## 1 Introduction

Varicella zoster virus (VZV) is responsible for two distinct clinical manifestations: primary varicella infection (chickenpox) and herpes zoster (HZ). Varicella typically occurs during the initial infection with VZV, most commonly in children [1, 2]. As the primary varicella infection resolves, the virus remains latent in the posterior spinal root ganglion or cranial ganglion and can later reactivate to cause HZ, due to immunosuppression or an age‐related reduction in immunity [3]. As one of the most common serious complications of HZ, post‐herpetic neuralgia (PHN) can lead to debilitating nerve pain that may persist for several months after the rash, particularly in elderly individuals [4]. Notably, three to five people among one thousand were reported to develop HZ per year worldwide, and the incidence rate is increasing annually. Therefore, HZ has significant impacts on global health and imposes a substantial social burden [5, 6]. Vaccination against HZ has become a critical component of the global strategy for preventing VZV infection.

The viral envelope of VZV particles is adorned with a range of glycoproteins, including gB, gC, gE, gH, gI, gK, and gL [7]. Notably, gE is the most prevalent glycoprotein and is integral to the processes of viral replication and transmission. Several critical domains and sites contribute to its functionality as an optimal antigen. The amino acids 539‐559, comprising the transmembrane domain, along with the glycosylation sites A_568_YRV_571_, Y_582_AGL_585_, and S_593_ES_595_T_596_DT_598_, are vital for transport from the endoplasmic reticulum (ER) to the *trans*‐Golgi network (TGN) in both infected and gE‐transfected cells [8]. Moreover, it is reported that the gE protein can elicit both humoral and cellular immune responses [9]. Among these, cell‐mediated immunity (CMI) is critical for preventing the reactivation of latent VZV. Clinical evidence indicates that stronger VZV‐CMI correlates with a decreased incidence and severity of HZ [10, 11, 12, 13, 14]. Specifically, the role of CD4^+^ T cells is critical for the resolution of VZV infections and serves as a key indicator for assessing the immunogenicity of vaccines [15].

Currently, four vaccines are approved for HZ prevention. SkyZoster (SK Bioscience), Zostavax (Merck) and Ganwei (Bchtpharm) are all live attenuated varicella vaccines. SkyZoster has only been authorized in countries such as South Korea, Thailand and Malaysia, and there is a lack of crucial clinical data on indicators such as its protective effect. Zostavax and Ganwei exhibits a protection efficacy of nearly 64% in individuals aged 60 to 69 years [16, 17]. Shingrix, which is developed by GSK, is a subunit protein vaccine adjuvanted with AS01B, a formulation that includes QS21, monophosphoryl lipid A, and liposomes [18]. This vaccine has a sustained and high efficacy rate of 97.4% in preventing HZ in individuals aged 60 to 69 years, with efficacy exceeding 90% across all tested age groups, including those aged 80 years and older. However, the significant reactogenicity of Shingrix and the high cost of the component QS21 limit its broader utilization [19, 20]. Considering the limitations of the approved vaccines, a new generation of vaccine technology, mRNA vaccines are expected to lead to a breakthrough in the development of HZ vaccines.

First, compared with the traditional vaccines, mRNA vaccines have simple production process without expression and purification of antigen protein, expediting development timelines. Second, mRNA vaccines exhibit no integration to the host genome and degrade in vivo, achieving an enhanced safety profile. Most importantly, mRNA vaccines have been widely reported to elicit both robust humoral and cellular immune responses [21]. To date, great efforts have been made for the development of mRNA vaccine against VZV infection. An mRNA vaccine encoding the VZV gE antigen was reported to elicit immunogenicity comparable to an adjuvanted subunit vaccine and superior to a live attenuated vaccine in non‐human primate models [22]. In another work, an mRNA vaccine platform exhibited superior immunogenicity and safety profiles compared to Shingrix in both mice and rhesus macaques; the ability of this vaccine to induce robust T‐cell immunity was particular strong [11]. Furthermore, different coding sequences of the gE protein has impacts on immunogenicity. An LNP‐mRNA vaccine prepared from the gE protein with a double‐mutated carboxyl terminus exhibited immunological efficacy comparable to that of Shingrix [23]. A particular full‐length gE antigen‐encoding mRNA candidate induced stronger immune responses and less inflammation than did Shingrix, surpassing its truncated variants with various mutations [24]. A VZV mRNA candidate vaccine was produced after C‐terminal mutations, optimization of the VZV YC03 strain gE sequence, and UTRs inserted into the gE, demonstrating comparable humoral immunogenicity and superior cellular immunogenicity to Shingrix and Zostavax in mice [25]. In addition, a highly stable lyophilized mRNA vaccine for HZ was reported to induce high levels of antibodies and robust CD4^+^ but also CD8^+^ immune responses along with long term stability [26]. However, despite of these advantages of mRNA vaccine, the long‐lasting protective capacity was rarely considered previously, which is critical for immune efficacy of vaccines.

Therefore, our work is aiming at developing a potent mRNA vaccine possessing long‐lasting protection. As reported, Transformer‐based deep learning models demonstrated exceptional capabilities for antiviral agent design [27, 28]. On this basis, to accelerate the development progress and elevate successful rate, artificial intelligence (AI) technology was utilized instead of experimental methods in the screening process of sequences. First, a variety of novel antigen sequences were artificially designed and several candidate variants were obtained through AI‐assiated screening. A comparative evaluation in terms of immunogenicity in mice were then conducted among these variants and several naturally highly expressed 5' untranslated region (UTR) sequences were further optimized. The specific full length mRNA sequence was delivered in a lipid nanoparticle (LNP), which successfully delivered SARS‐CoV‐2 vaccine in our recent work [29]. Thereafter, we assessed the immunization interval, dose, and effectiveness of the mRNA‐LNP vaccine in inducing VZV‐specific humoral responses and CMI in mice and rhesus macaques. Our results proved that the mRNA‐LNP vaccine (VZV‐gEmD) had significant benefits in terms of gE‐specific IgG titers and T‐cell responses. Compared with the Shingrix recombinant subunit vaccine, this vaccine demonstrates superior immunogenicity and immunological persistence, with a robust cellular immune response that persists for more than one year. These findings suggest that our mRNA vaccine has significant potential for development as a more effective HZ vaccine.

## 2 Results

### 2.1 Predictive Modeling of Functional Properties in VZV gE Variants

**FIGURE 1:**
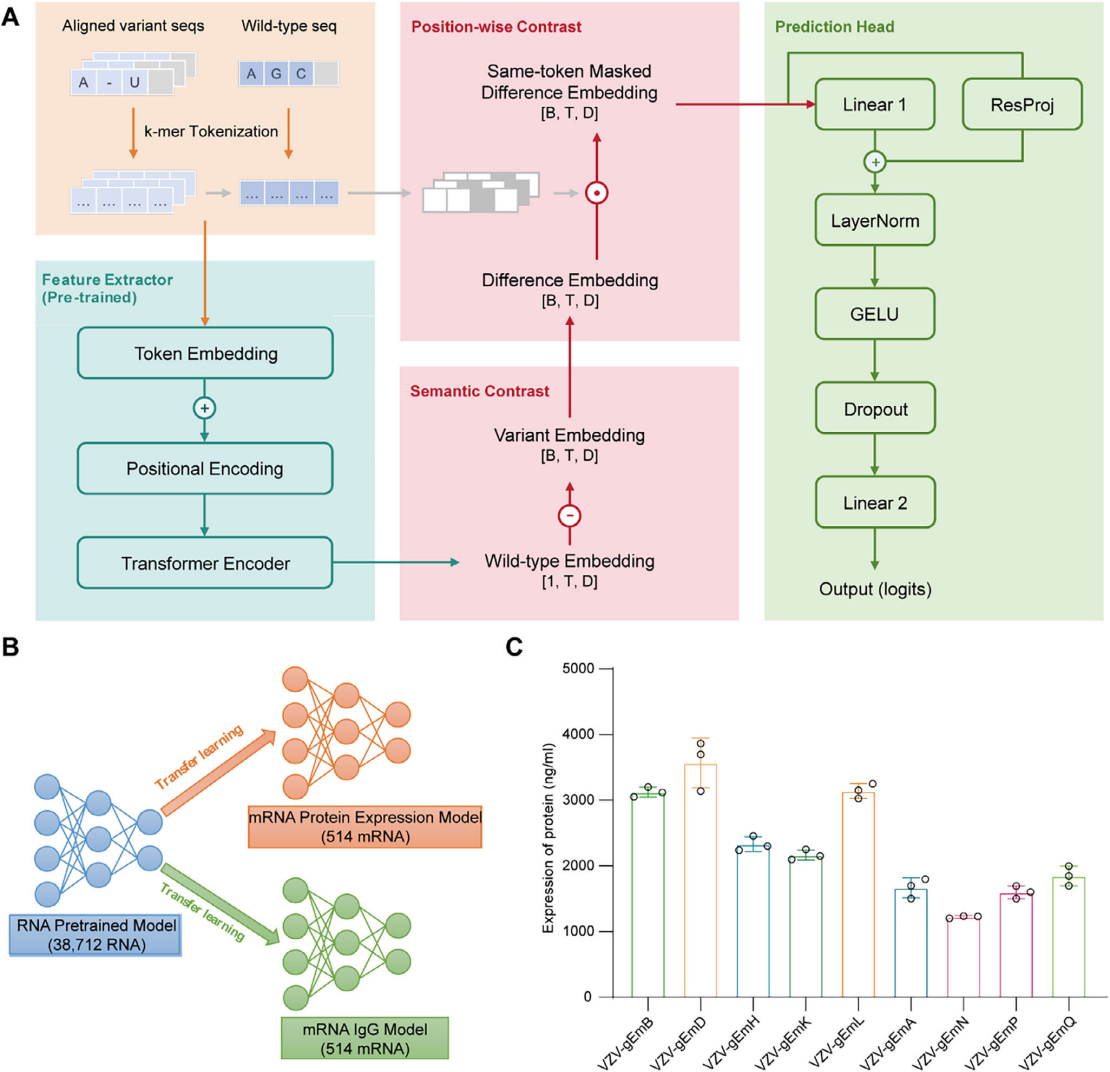
Artificial intelligence‐assisted design of mRNA antigen sequences. (A) Architecture of the proposed RDTransformer for fine‐tuning tasks. The model is built upon a Transformer backbone and introduces a dynamic difference‐embedding scheme computed relative to the wild‐type sequence, which suppresses noise at non‐mutated positions and selectively amplifies the representational impact of introduced mutations. (B) Transfer learning strategy for the proposed model: pre‐training on 38 712 ncRNA sequences, with subsequent fine‐tuning to predict protein expression and IgG levels separately. (C) Expression levels of gE protein encoded by the mRNA vaccine candidates (n=3) in HEK293T cells assessed by enzyme‐linked immunosorbent assay (ELISA). Data are represented as mean ± SEM.

During the development of VZV vaccines, 514 VZV gE protein sequences were developed based on manual experience. Their protein expression levels and antibody responses were evaluated and the data were used to train and develop an AI prediction model capable of screening sequences with both high protein expression and strong IgG antibody reactivity (Figure 1A,B).

#### 2.1.1 Convergence and Downstream Effectiveness of Pre‐Training

**FIGURE 2:**
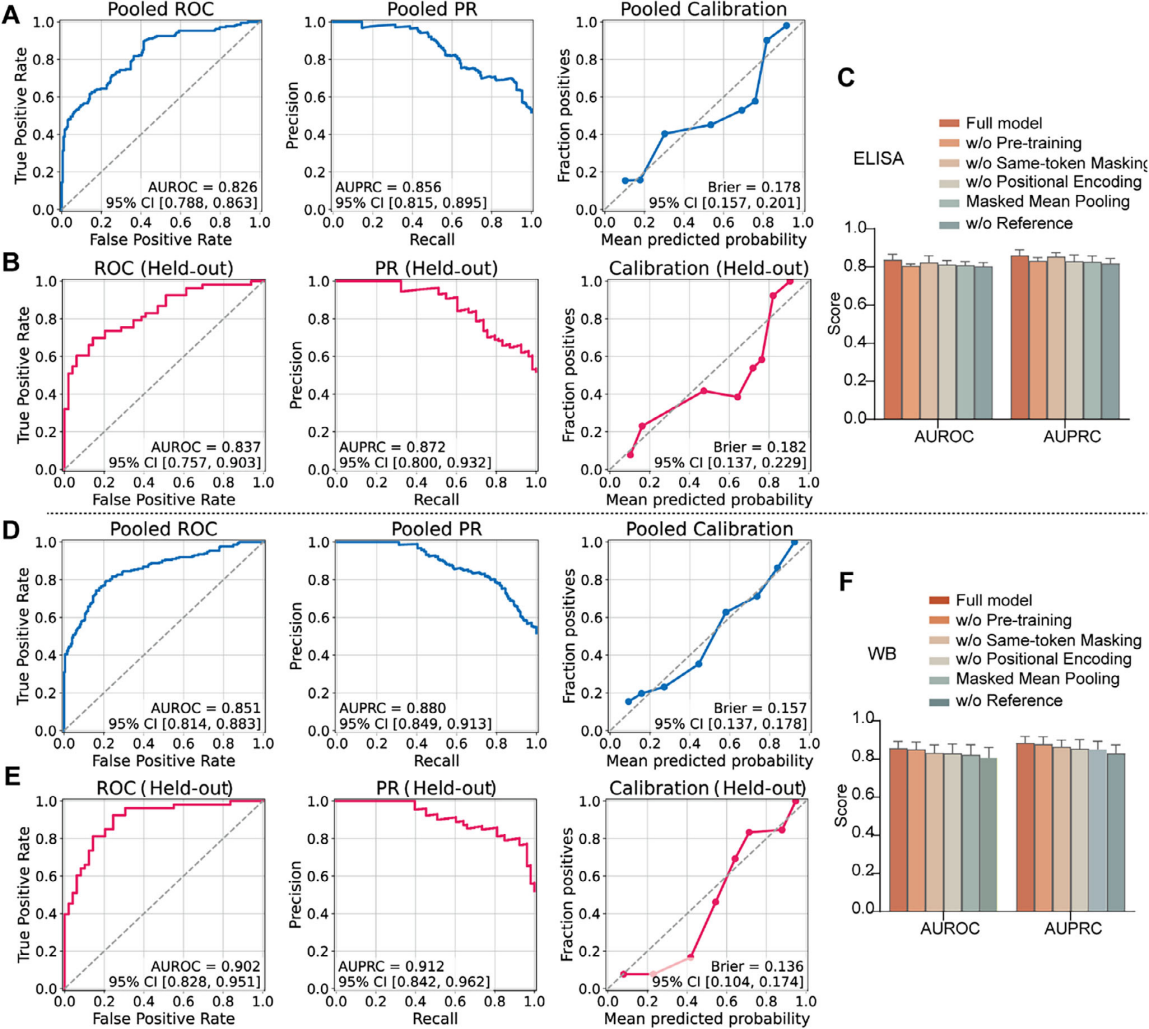
Performance evaluation and ablation analysis of the fine‐tuned model. Performance of the IgG level prediction model on the pooled cross‐validation predictions (A) and on the held‐out test set (B). (C) Mean discrimination performance of the ablated model variants across cross‐validation folds. Performance of the protein expression prediction model on the pooled cross‐validation predictions (D) and on the held‐out test set (E). (F) Mean discrimination performance of the ablated model variants across cross‐validation folds. Data are represented as mean ± SD.

To ensure that the pre‐training effectively captured generalizable RNA sequence representations, we monitored the convergence of training and validation curves. The training loss steadily decreased while evaluation metrics (AUROC and F1‐score) increased, with both trends ultimately stabilizing and exhibiting a minimal generalization gap, indicating robust learning without overfitting (Figure S1A). Early stopping was triggered at epoch 52, and the weights from the best‐performing epoch (epoch 44) were restored. This checkpoint achieved a training loss of 0.360 and a validation loss of 0.357, corresponding to a generalization gap of 0.003. The model's discriminative ability was further supported by a high validation AUROC of 0.987 and a confusion matrix showing predominantly correct classifications (Figure S1B). The effectiveness of pre‐training was quantified through an ablation study on the training set. As demonstrated by 4‐fold cross‐validation, the pre‐trained model substantially outperformed a model trained from scratch in the IgG level prediction task, achieving a mean AUROC of 0.837 (vs. 0.806) and a mean AUPRC of 0.861 (vs. 0.832) (Figure 2E,F). Collectively, these results indicate that the training procedure successfully produced a model with strong generalization potential for downstream tasks.

#### 2.1.2 Cross‐Validation Performance

To assess the stability and generalizability of the fine‐tuned models, 4‐fold cross‐validation was performed on the training set. The IgG level prediction model attained a mean AUROC of 0.837, and the protein expression prediction model achieved a mean AUROC of 0.856. Both models also exhibited good calibration, as reflected by their well‐aligned calibration curves and low Brier scores (Table S1). In addition to the fold‐wise results, performance metrics were calculated for the pooled predictions across all folds. Consistent with the fold‐wise outcomes, both models demonstrated robust discrimination, with pooled AUROCs of 0.826 for the IgG level prediction model and 0.851 for the protein expression model (Figure 2A,D). Learning curves further indicated stable convergence without notable overfitting for either model (Figure S2).

#### 2.1.3 Held‐Out Test Set Performance

On the independent hold‐out test set, the final models exhibited strong performance. The IgG level prediction model achieved an AUROC of 0.837, and the protein expression prediction model reached an AUROC of 0.902. Both models demonstrated robust discriminative ability and well‐aligned calibration curves (Figure 2B,E).

#### 2.1.4 Ablation Study of Key Model Components

Ablation analyses further highlighted the contributions of key model components. Removing the wild‐type reference sequence input resulted in the largest decrease in mean AUROC across folds (IgG level: −0.034; protein expression: −0.048). Similarly, excluding position‐wise same‐token masking substantially impaired performance (IgG level: −0.013; protein expression: −0.024). The removal of either pre‐training or positional encoding also led to considerable performance drops, whereas max pooling consistently outperformed mean pooling (Figure 2C,F). Furthermore, these findings were corroborated by evaluation metrics computed on predictions pooled from all folds (Figure S3; Tables S2 and S3).

### 2.2 Screening and Production of mRNA Vaccine Candidates

A variety of mRNA variants featuring distinct mutations were designed on the basis of the sequence that encodes glycoprotein gE of the VZV Oka strain. The gE transmembrane protein features a long N‐terminal extracellular domain, a brief transmembrane domain, and a short C‐terminal cytoplasmic domain. Studies have demonstrated that the C‐terminal domain is pivotal for the localization of the gE protein [30, 31]. Different modifications of the gE antigen, including full‐length gE, its truncated variants, and C‐terminal mutations, can affect its immune stimulation effect [32, 33]. Therefore, we conducted an empirical design of mRNA candidates encoding the gE antigen, focusing on this functional domain.

To augment the immunogenicity of VZV gE, rational design strategies was implemented, encompassing: (i) combinatorial mutagenesis of key residues or sequence deletion engineering targeting loci that modulate the intracellular trafficking machinery of gE among the endoplasmic reticulum (ER), TGN, and endosomes; (ii) site‐directed mutagenesis or C‐terminal truncation of gE to preserve critical functional motifs; and (iii) codon optimization of the antigenic sequence to enhance translational efficiency.

Subsequently, the sequences possessing high levels of protein expression and gE‐ specific IgG antibodies were predicted and identified as the vaccine candidates with the assistance of the AI model, including: (i) Full‐length gE variants (VZV‐gEmB, VZV‐gEmD, VZV‐gEmH, VZV‐gEmK, and VZV‐gEmL), produced by modification of the C‐terminal domain via single or multiple mutations. (ii) Truncated gE variants (VZV‐gEmN, VZV‐gEmP, and VZV‐gEmA), produced through the deletion of amino acids 574 to 623 in the C‐terminal domain, accompanied by a single point mutation. (iii) A truncated gE variant (VZV‐gEmQ), created via the deletion of amino acids 588 to 623 in the C‐terminal domain with a single point mutation. Furthermore, ten amino acids in the C‐terminal domain near the transmembrane region were successively deleted. In the generated mRNA molecules, all uracil (U) residues are substituted with N1‐methyl‐pseudouridine (N1‐PU) to increase mRNA stability and mitigate nonspecific immune responses.

Following the synthesis of the mRNA constructs, the mRNAs were purified and evaluated for several critical characteristics (Table S4). Subsequently, the mRNAs were encapsulated within YK‐009 lipid‐based lipid nanoparticles (LNPs), as previously described in detail [29]. The resulting mRNA‐LNP formulations exhibited a consistent particle size with a polydispersity index (PDI) below 0.2, indicating a high degree of uniformity among the nanoparticles (Table S5). The results from all the analytical tests conducted on the mRNA‐LNPs confirmed the successful formulation of the vaccines. Next, we successfully confirmed the expression of gE protein encoded by all nine mRNA vaccines in HEK293T cells through ELISA assay (Figure 1C). The protein expression levels varied across sequences, with higher levels observed for full‐length gE antigen sequences than for truncated sequences. These results confirmed the successful production and intracellular gE expression of the nine gE antigen variants.

### 2.3 Evaluation and Comparison of the Humoral and Cellular Immunogenicity of mRNA‐LNP Vaccine Candidates in BALB/c Mice

**FIGURE 3:**
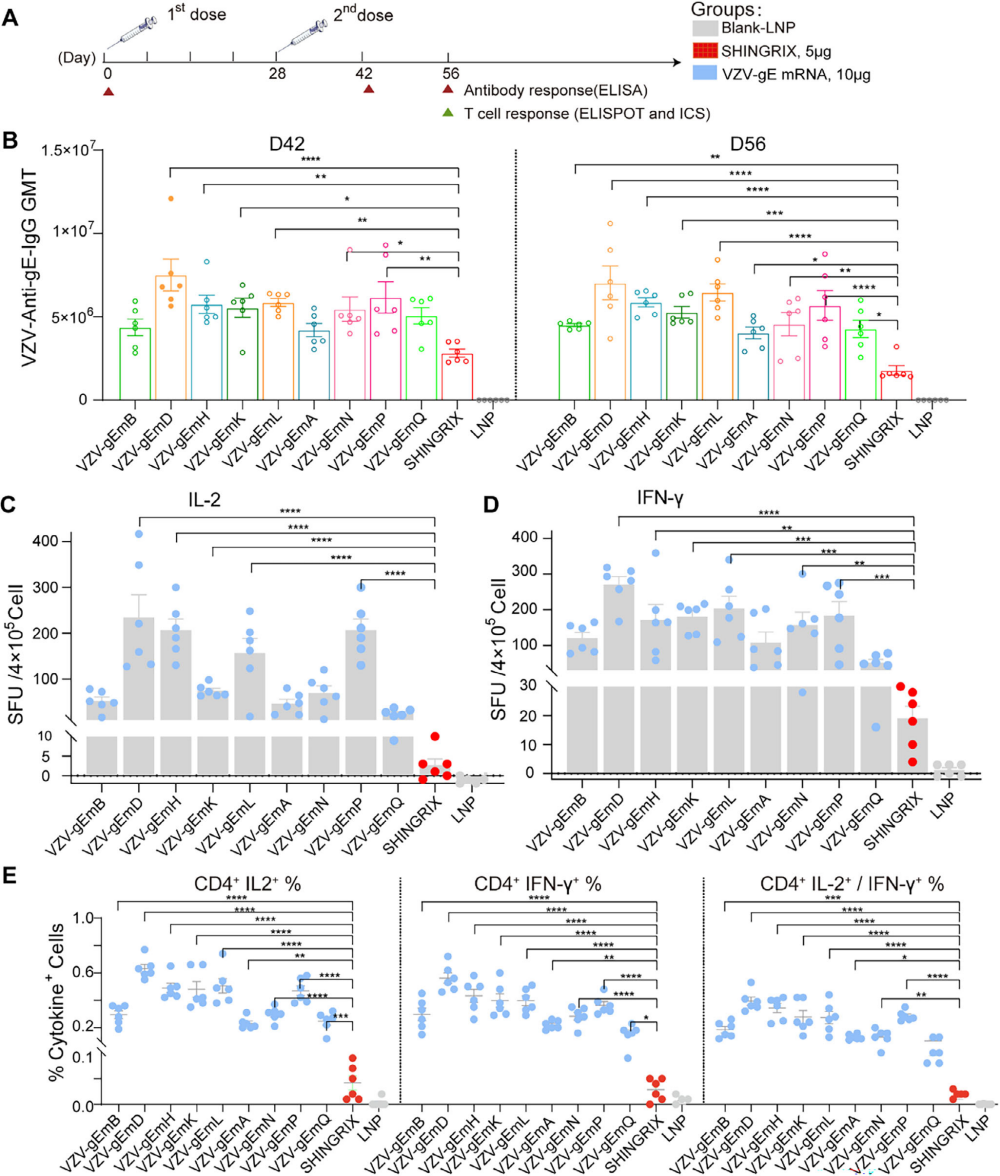
Evaluation and comparison of the humoral and cellular immunogenicity of mRNA‐LNP vaccine candidates in BALB/c mice. (A) Diagram of the vaccination and sample collection schedule. BALB/c mice (n = 6) were i.m. immunized twice with different vaccines at 10 µg on days 0 and 28. Blood samples were collected at the indicated time points. Spleens were collected 28 days after the second vaccination on day 56. (B) Quantification of VZV‐Anti‐gE‐IgG antibodies by ELISA in serum collected on days 42 and 56. Secreted levels of IL‐2 (C) and IFN‐γ (D) in splenocytes were assessed via enzyme‐linked immunospot (ELISPOT) Assay. (E) Proportions of gE‐specific IL‐2‐ and IFN‐γ‐producing CD4^+^ T cells detected by flow cytometry. Data are represented as mean ± SEM. One‐way ANOVA was used for statistical analysis. * *p* < 0.05, ** *p* < 0.01, *** *p* < 0.001, **** *p* < 0.0001.

Upon the successful development of all nine mRNA‐LNP formulations, we conducted a comparative analysis of the humoral and cellular immune responses elicited in mice to identify the most promising candidate vaccine (Figure 3A). For the antibody responses, all nine candidate vaccines elicited elevated levels of gE‐specific IgG antibodies, significantly surpassing those observed in the Shingrix group (Figure 3B, *p* < 0.05). Notably, the full‐length antigens VZV‐gEmD and VZV‐gEmL and the truncated variant VZV‐gEmP exhibited superior immunogenicity. Next, the induction of gE‐specific CD4^+^ T cells was assessed, as the CD4^+^ T‐cell immune response is recognized as a critical parameter for assessing the immune efficacy of vaccines, particularly in the context of VZV infection and vaccine‐induced immunity [12]. Their production of IFN‐γ and IL‐2 was detected via an enzyme‐linked immunospot (ELISPOT) assay at 28 days post‐second immunization. Compared with the Shingrix vaccine, all the candidate mRNA vaccines elicited significantly elevated levels of the cytokines IFN‐γ and IL‐2 (*p* < 0.01). Specifically, the VZV‐gEmD group presented the highest cytokine levels, though there was no significant difference between VZV‐gEmD and part of the other candidate vaccines (Figure 3C,D; Table S6). Additionally, the percentages of IL‐2 or/and IFN‐γ‐producing CD4⁺ T cells in all the candidate vaccine groups were also significantly higher than those in the Shingrix group. The VZV‐gEmD group again demonstrated the most pronounced immunogenicity regarding these cell percentages (Figure 3E). These results clearly demonstrated the advantage of mRNA vaccines over traditional vaccines in inducing robust T‐cell immunity. Therefore, owing to the enhanced potency in eliciting both humoral and cellular immune responses, VZV‐gEmD was identified as the most promising candidate for further investigation in vaccine development.

### 2.4 The cellular Localization of gE Antigen Candidates is Correlated With Vaccine Immunity

**FIGURE 4:**
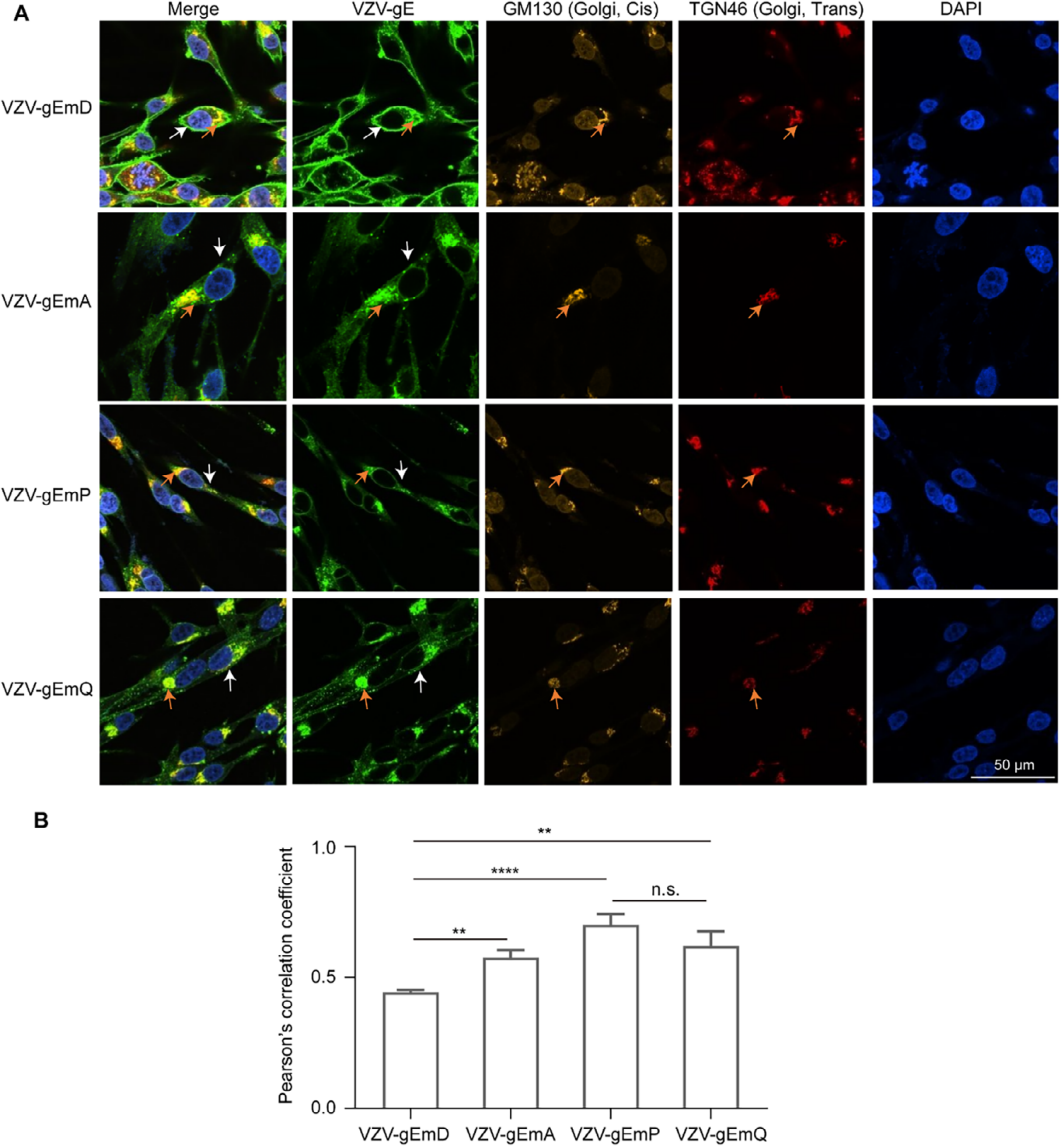
The cellular localization and expression of antigen candidates in MeWo cells. (A) Confocal microscopic images depicting the subcellular localization of VZV gE protein in the Golgi apparatus. (B) Colocalization analysis of VZV gE protein with trans‐Golgi network for mRNA constructs, quantified by Pearson's correlation coefficient. gE antigen: green; *Cis*‐Golgi: orange; *Trans*‐Golgi: red; DAPI: blue. The white arrows point to the cell membrane. The orange arrows indicate the location of the Golgi. Scale bar: 50 µm. Data are represented as mean ± SEM. One‐way ANOVA was used for statistical analysis. ** *p* < 0.01, **** *p* < 0.0001.

To confirm the possible contribution of cellular localization to the potent immunogenicity of VZV‐gEmD, we observed the locations of various antigens in cells via laser confocal microscopy. As reported, gE undergoes high‐mannose oligosaccharide modification in the endoplasmic reticulum, then traverses the Golgi apparatus for further modifications, and is finally transported directly to the cell membrane [34]. This process is highly dynamic: the membrane‐anchored gE protein can be further internalized from the plasma membrane via endocytosis, recycled to the *trans*‐Golgi network (TGN) by endosomes, and subsequently shuttled back to the plasma membrane [8]. The intracellular C‐terminal domain of the gE protein includes critical phosphorylation sites and sequences that facilitate targeting to the TGN, which significantly influences the intracellular transport and immunogenic properties of gE [30, 31, 35]. Our modifications to the gE sequences also focused on this region. The localization experiment incorporated four samples, the full‐length sequence VZV‐gEmD, the truncated sequences VZV‐gEmP and VZV‐gEmA, and the truncated sequence with an extra deletion, VZV‐gEmQ. As indicated, all the samples accumulated within the Golgi apparatus and cell membrane (Figure 4; Figure S4), which is highly consistent with the previously reported maturation and trafficking route of the gE protein. Notably, the full‐length antigen VZV‐gEmD demonstrated the strongest localization within the cell membrane (Figure 4; Figure S4), which could be attributed to enhanced gE trafficking and surface expression due to sequence modification [24]. Furthermore, precisely due to its robust transport to the cell membrane, its accumulation in the TGN is relatively low. In contrast, the other three truncated sequences exhibited lower localization in the cell membrane along with enhanced accumulation in TGN (Figure 4). The colocalization levels between gE and the TGN, indicated by Pearson's correlation coefficient in Mewo cells, aligned with these observations. Moreover, as indicated by our assessment, the full‐length sequence VZV‐gEmD, which is located mostly in the cell membrane, induced the most potent immunogenicity among these tested sequences (Figure 3). These observations suggest that antigen localization in the cell membrane may result in better antigen presentation and promote humoral and cellular immune responses.

### 2.5 Immunogenicity of mRNA‐LNP Vaccines With Different 5’UTR Sequences

**FIGURE 5:**
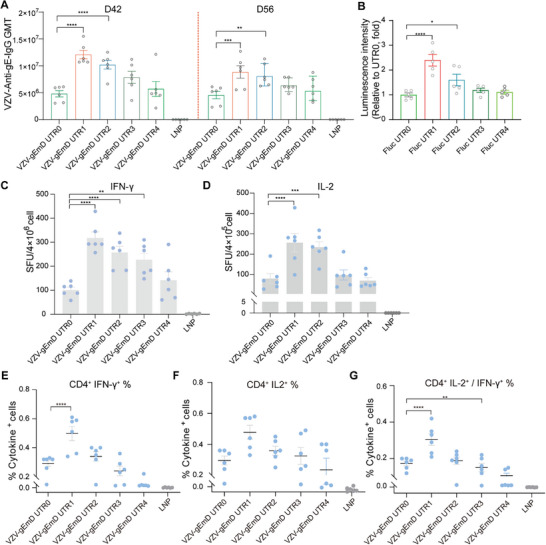
The immunological efficacy of mRNA‐LNP vaccines with different 5’UTR sequences in BALB/c mice (n=6). (A) Quantification of VZV‐Anti‐gE‐IgG antibodies in different vaccine groups by ELISA in serum collected on days 42 and 56. (B) Assessment of protein expression levels of sequences bearing different 5’UTRs. Secreted levels of IFN‐γ (C) and IL‐2 (D) in splenocytes were assessed by ELISPOT assay. (E)(F)(G) Proportions of gE‐specific IL‐2‐ and IFN‐γ‐producing CD4^+^ T cells detected by flow cytometry. Data are represented as mean ± SEM. One‐way ANOVA was used for statistical analysis. ** *p* < 0.01, *** *p* < 0.001, **** *p* < 0.0001.

To optimize our mRNA‐LNP vaccines regarding their 5’UTR sequences, both natural and modified 5' UTRs of highly expressed genes in humans were screened via the immunogenicity in BALB/c mice. As a structural component of mRNA vaccine candidates, the 5'UTR, is instrumental in modulating the translation of mRNAs into proteins, influencing the stability, localization, and overall translational efficiency of the mRNA molecules [36]. In this evaluation, the antigen sequence utilizing UTR0 (Gene ID: 3039, accession: NM_000558), which is the same UTR employed in the COVID‐19 mRNA vaccine (BNT162b2) [37], served as a positive control group. The blank LNP group served as a negative control. The minimum free energy (MFE) predictions of all tested UTRs are summarized in Table S7. For humoral immunity, all the candidate vaccines induced comparable levels of anti‐gE IgG specific antibodies to the UTR0‐bearing sequence. Among them, the UTR1 and UTR2 groups presented significantly higher levels of anti‐gE IgG antibodies than did the UTR0 group (Figure 5A, *p* < 0.01). Importantly, protein expression levels in these groups followed the same trend, with the UTR1 and UTR2 constructs exhibiting markedly higher expression than UTR0 (Figure 5B). This consistency suggests that enhanced protein expression driven by optimized 5’UTRs may serve as a potential mechanism underlying the improved humoral immunogenicity observed. For more critical cellular immunity, the levels of IFN‐γ and IL‐2 in response to the candidate vaccines containing UTR1 and UTR2 were significantly elevated relative to those in the UTR0 group (Figure 5C,D, *p* < 0.001), along with an increase of the proportions of CD4^+^IFN‐γ^+^, CD4^+^IL‐2^+^, and CD4^+^IFN‐γ^+^IL‐2^+^ cells (Figure 5E–G; Figure S5). Notably, there was a significant increase in the size of CD4^+^IFN‐γ^+^ and CD4^+^IFN‐γ^+^IL‐2^+^ populations in the UTR1 group compared with those in the UTR0 control group (Figure 5E,F, *p* < 0.0001). Consequently, we chose UTR1 as the 5' UTR element for the vaccine candidate. Collectively, screening UTRs further enhances the candidate vaccine's ability to induce more potent cellular immunity, which is critical for VZV prevention.

### 2.6 The Immunogenicity of VZV‐gEmD Injection at Different Immunization Intervals in BALB/c Mice

**FIGURE 6:**
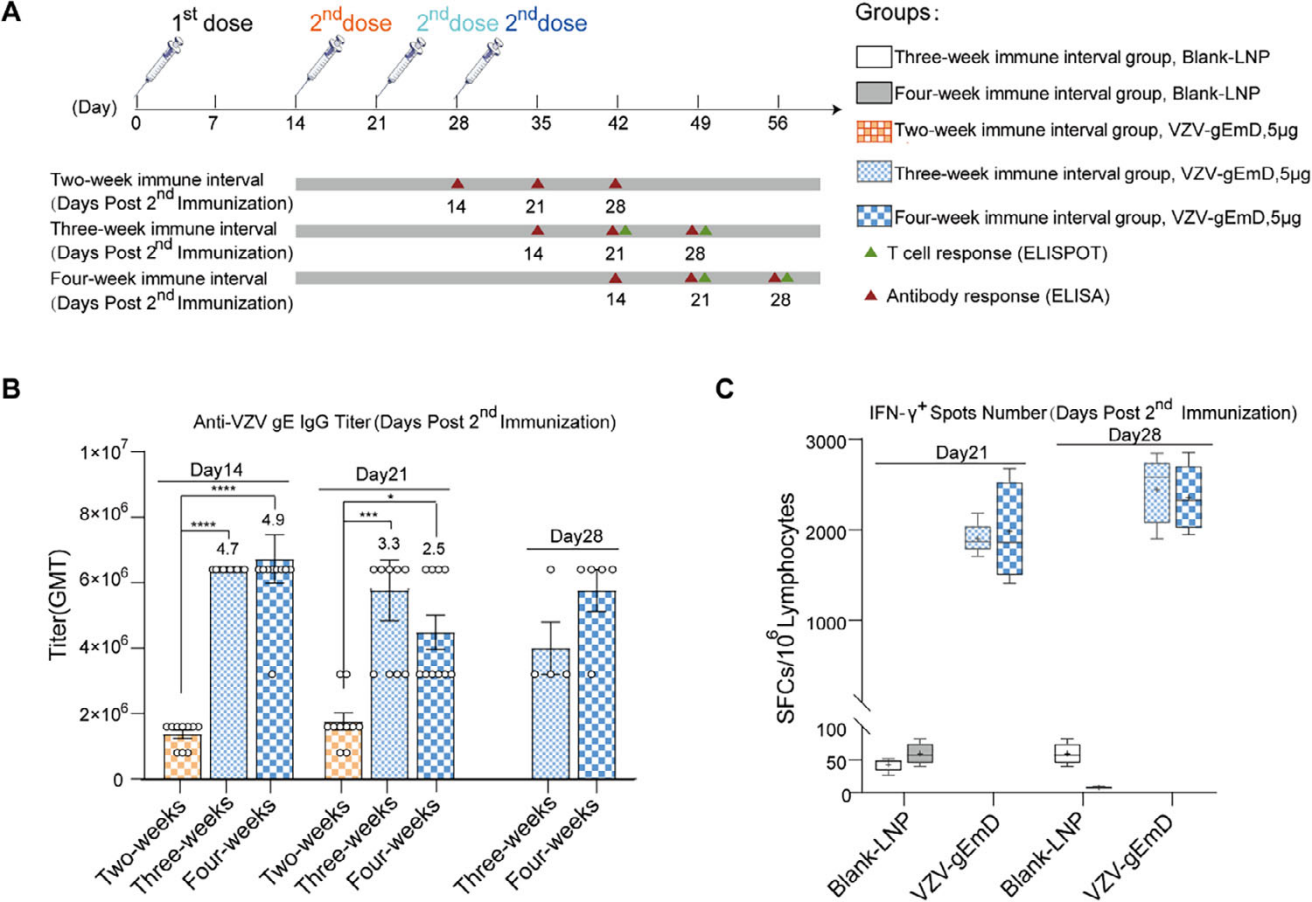
The immunological efficacy of VZV‐gEmD injection at different immunization intervals in BALB/c mice. (A) Schematic diagram of the vaccination and sample collection schedule. BALB/c mice were i.m. immunized twice at different immunization intervals of two, three, and four weeks. Blood samples were collected at the indicated time points. Spleens in groups of 3‐ and 4‐week interval were collected 21 and 28 days after the second vaccination. (B) Quantification of VZV‐Anti‐gE‐IgG antibodies by ELISA in serum collected on days 14, 21 and 28 (n = 10). (C) Secreted levels of IFN‐γ in splenocytes were assessed via ELISPOT assay (n = 5). Data are represented as mean ± SEM. One‐way ANOVA was used for statistical analysis. * *p* < 0.05, *** *p* < 0.001, **** *p* < 0.0001. The numbers above the bars represent the multiple of the average value compared to that of the group with 2‐week interval.

To assess the immune responses elicited by VZV‐gEmD at various immunization intervals, which could inform the selection of appropriate intervals for clinical trials in humans, we administered two intramuscular injections of VZV‐gEmD to BALB/c mice at three different intervals: two weeks, three weeks, and four weeks (Figure 6A). The gE‐specific IgG antibody titers elicited by VZV‐gEmD in the groups with 3‐week and 4‐week intervals were significantly elevated compared with those in the 2‐week interval group (*p* < 0.05). The 4‐week interval group maintained a slightly higher immunogenicity than the 3‐week group until day 28, which is not statistical (Figure 6B). This finding indicates that suitable interval time contributed to high immunogenicity. Next, for cellular immunity evaluation, we further quantified the number of IFN‐γ+‐secreting splenocytes in the 3‐ and 4‐week groups (Figure 6C). Significant VZV gE‐specific cellular immune responses were activated following the administration of the VZV‐gEmD vaccine, regardless of whether a 3‐week or 4‐week immunization interval was employed. The cellular immune responses were robust on day 21 and maintained a high level until day 28. In summary, VZV‐gEmD injection effectively elicited substantial and enduring humoral and cellular immune responses, particularly when the 4‐week immunization interval was used.

### 2.7 The Immunogenicity of VZV‐gEmD after Repeated Intramuscular Injection in BALB/c Mice

**FIGURE 7:**
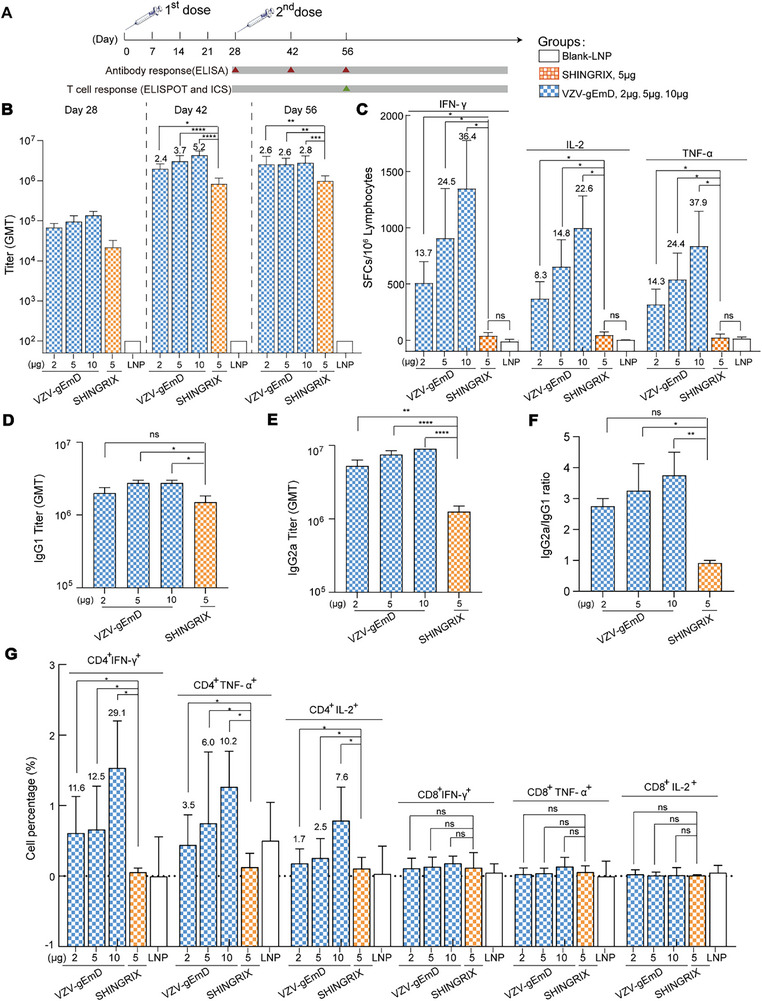
The immunogenicity of VZV‐gEmD injection at various doses in BALB/c mice. (A) Schematic diagram of the vaccination and sample collection schedule. BALB/c mice (n = 6) were i.m. immunized twice (day 0 and day 28) with blank‐LNP, Shingrix, or VZV‐gEmD at various doses. Blood samples were collected at the indicated time points. Spleens were collected 28 days after the second vaccination on day 56. (B) Quantification of VZV‐Anti‐gE‐IgG antibodies by ELISA in serum collected on days 28, 42 and 56. (C) Secreted levels of IFN‐γ, IL‐2, and TNF‐α in splenocytes assessed via the ELISPOT assay. Measurement of gE‐specific‐IgG1 (D) and IgG2a (E) titer levels and IgG2a/ IgG1 ratio (F) by ELISA assay. (G) Proportions of gE‐specific IFN‐γ‐, TNF‐α‐ and IL‐2‐producing CD4^+^ and CD8^+^ T cells detected by flow cytometry. Data are represented as mean ± SEM. One‐way ANOVA was used for statistical analysis. n.s., not significant; * *p* < 0.05; ** *p* < 0.01; *** *p* < 0.001; **** *p* < 0.0001. The numbers above the bars represent the multiple of the average value relative to that of the Shingrix group.

To validate the potent immunogenicity of VZV‐gEmD injection, VZV‐specific immune responses were evaluated in BALB/c mice. Shingrix and blank LNP groups served as positive and negative controls, respectively (Figure 7A). Antibody responses were measured every 2 weeks after the second immunization. Results showed that all the VZV‐gEmD groups elicited gE‐specific IgG antibodies with titers surpassing those observed in the Shingrix group at all tested time points. The antibody titers in VZV‐gEmD groups increased over time (Figure 7B). Furthermore, the immunoglobulin subclasses IgG1 and IgG2a were analyzed to evaluate Th1/Th2 polarization [38, 39]. Immunization of BALB/c mice with VZV‐gEmD induced the production of gE‐specific IgG1 and IgG2a antibodies, with the levels of both subclasses significantly exceeding those induced by the positive control, Shingrix (Figure 7D,E). Moreover, in contrast to Shingrix, which tended to induce a Th2‐biased IgG response (IgG1), VZV‐gEmD exhibited a greater tendency to elicit a Th1‐biased IgG response (IgG2a), as evidenced by a higher IgG2a/IgG1 ratio (Figure 7F). More importantly, we evaluated the cytokine secretion and the proportion of relevant T cells in mice for cellular immunity assessment. All three VZV‐gEmD dosages (2, 5, 10 µg/mouse) induced significantly stronger T‐cell responses than Shingrix group (5 µg/mouse, 1/10 of human dose). Specifically, at 4 weeks post‐secondary immunization (D56), the secretion of IFN‐γ, IL‐2, and TNF‐α showed a dose‐dependent increase relative to the positive control Shingrix group: 13.7‐, 8.3‐, and 14.3‐fold higher in the low‐dose group (2 µg/mouse), 24.5‐, 14.8‐, and 24.4‐fold higher in the 5 µg/mouse group, and 36.4‐, 22.6‐, and 37.9‐fold higher in the high‐dose group (10 µg/mouse) (Figure 7C, *p* < 0.05). Similarly, the proportions of IFN‐γ⁺CD4⁺, TNF‐α⁺CD4⁺, and IL‐2⁺CD4⁺ cells in the low‐dose (2 µg/mouse), middle‐dose (5 µg/mouse), and high‐dose groups (10 µg/mouse) were 11.6‐, 3.5‐, and 1.7‐fold; 12.5‐, 6.0‐, and 2.5‐fold; and 29.1‐, 10.2‐, and 7.8‐fold higher than those in the positive control Shingrix group, respectively (Figure 7G; Figures S6 and S7, *p* < 0.05). Conversely, the proportions of CD8^+^IFN‐γ^+^, CD8^+^TNF‐α^+^, and CD8^+^IL‐2^+^ cells in all the test groups were not significantly different from those in the LNP control group (Figure 7G, *p* > 0.05).

**FIGURE 8:**
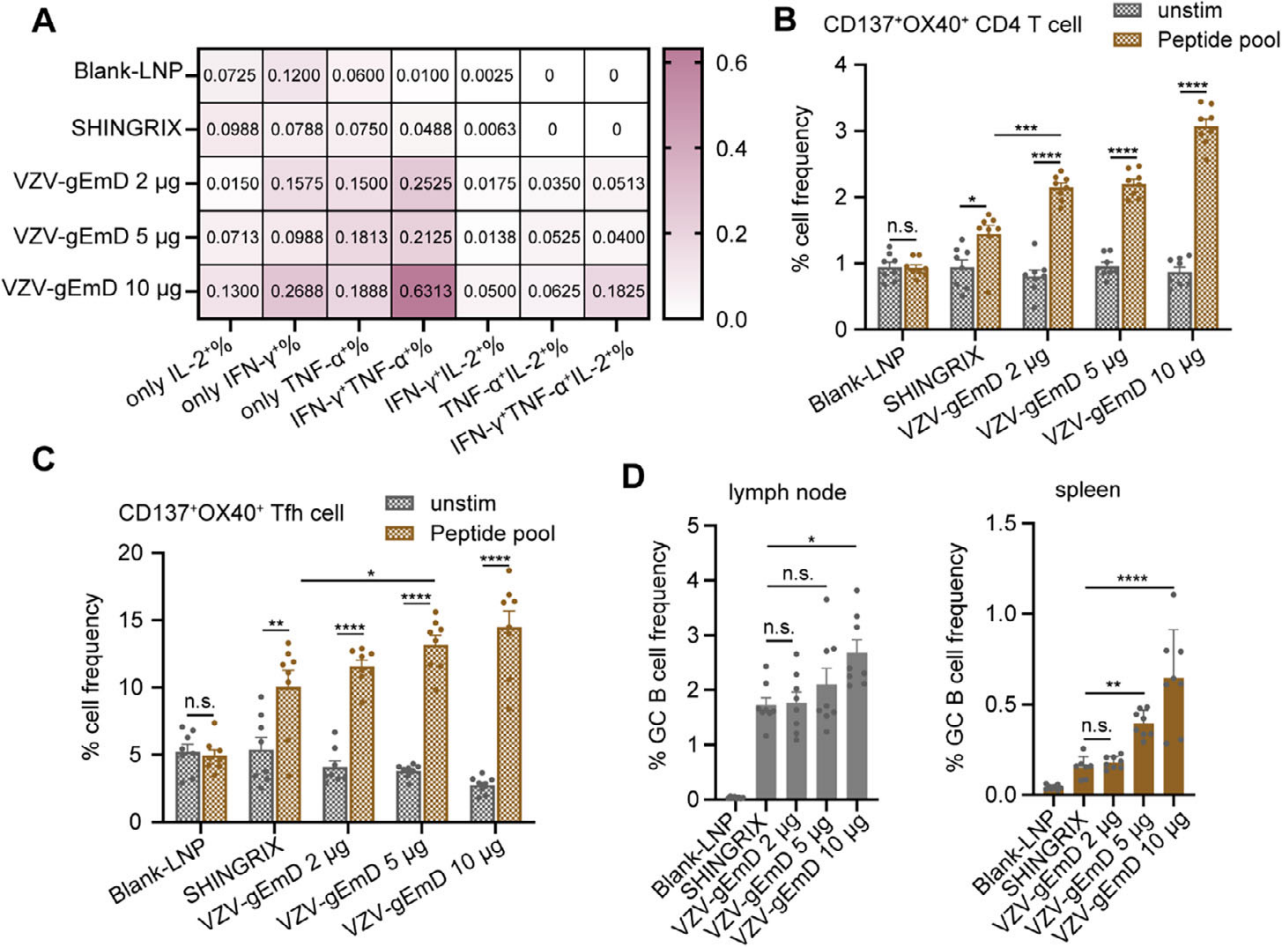
Comprehensive analysis of CD4⁺ T cell multifunctionality, Tfh activation, and germinal center B cell responses induced by VZV‐gEmD injections. (A) Cell frequency of single‐, double‐, and triple‐positive subsets for IFN‐γ, IL‐2, and TNF‐α in response to different VZV‐gEmD doses (2, 5, 10 µg) and controls (Blank‐LNP, Shingrix). Frequency of CD137⁺OX40⁺ activated CD4⁺ T cells (B) and Tfh cells (C) in response to VZV‐gEmD injections. (D) Frequency of germinal center B cells (GC B cells) in lymph nodes and spleens. Data are represented as mean ± SEM. T‐test and One‐way ANOVA were used for statistical analysis. n.s., not significant; * *p* < 0.05; ** *p* < 0.01; *** *p* < 0.001; **** *p* < 0.0001.

Multifunctional T cells serve as pivotal effector cells in the induction of protective immunity, and the present study undertook comprehensive characterization of their functional phenotypes. Analysis of cytokine secretion profiles demonstrated that T cells in the Shingrix group exhibited predominant single‐cytokine expression, whereas those in the VZV‐gEmD group displayed the capacity to secrete combinatorial cytokines, including dual‐cytokine and tri‐cytokine subsets (Figure 8A), indicative of expanded immunomodulatory versatility. Further interrogation of glycoprotein E‐specific T cell subsets revealed a significantly higher frequency of activation‐induced marker‐positive (AIM⁺, defined as CD137⁺OX40⁺) CD4⁺ T cells in the VZV‐gEmD LNP‐immunized cohort relative to the Shingrix group (Figure 8B), providing direct evidence for the enhanced CD4⁺ T cell activation potency of this vaccine candidate. Concomitantly, the frequency of CD137⁺OX40⁺ follicular helper T (Tfh) cells in the VZV‐gEmD group exhibited a statistically significant dose‐dependent elevation (Figure 8C). These T cell activation signatures were tightly correlated with the increased frequency of germinal center (GC) B cells (Figure 8D), as Tfh cells, a critical subset of CD4⁺ T cells, fulfill a central role in delivering helper signals to B cells and are indispensable for the initiation and sustenance of germinal center responses. Collectively, these findings suggest that the robust T cell activation elicited by VZV‐gEmD could augment Tfh cell functionality, thereby facilitating the proliferation and differentiation of GC B cells and endowing the candidate with the potential to induce long‐lasting antibody responses.

Overall, VZV‐gEmD, which was administered intramuscularly twice at 4‐week intervals in BALB/c mice, effectively induced both Th1‐biased cellular and humoral immune responses. Notably, under the currently established experimental conditions, the immunogenicity of this vaccine was superior to that of the positive control, Shingrix, with the 10 µg dosage demonstrating the most pronounced responses.

### 2.8 The Immunogenicity of VZV‐gEmD Injection in Rhesus Macaques

**FIGURE 9:**
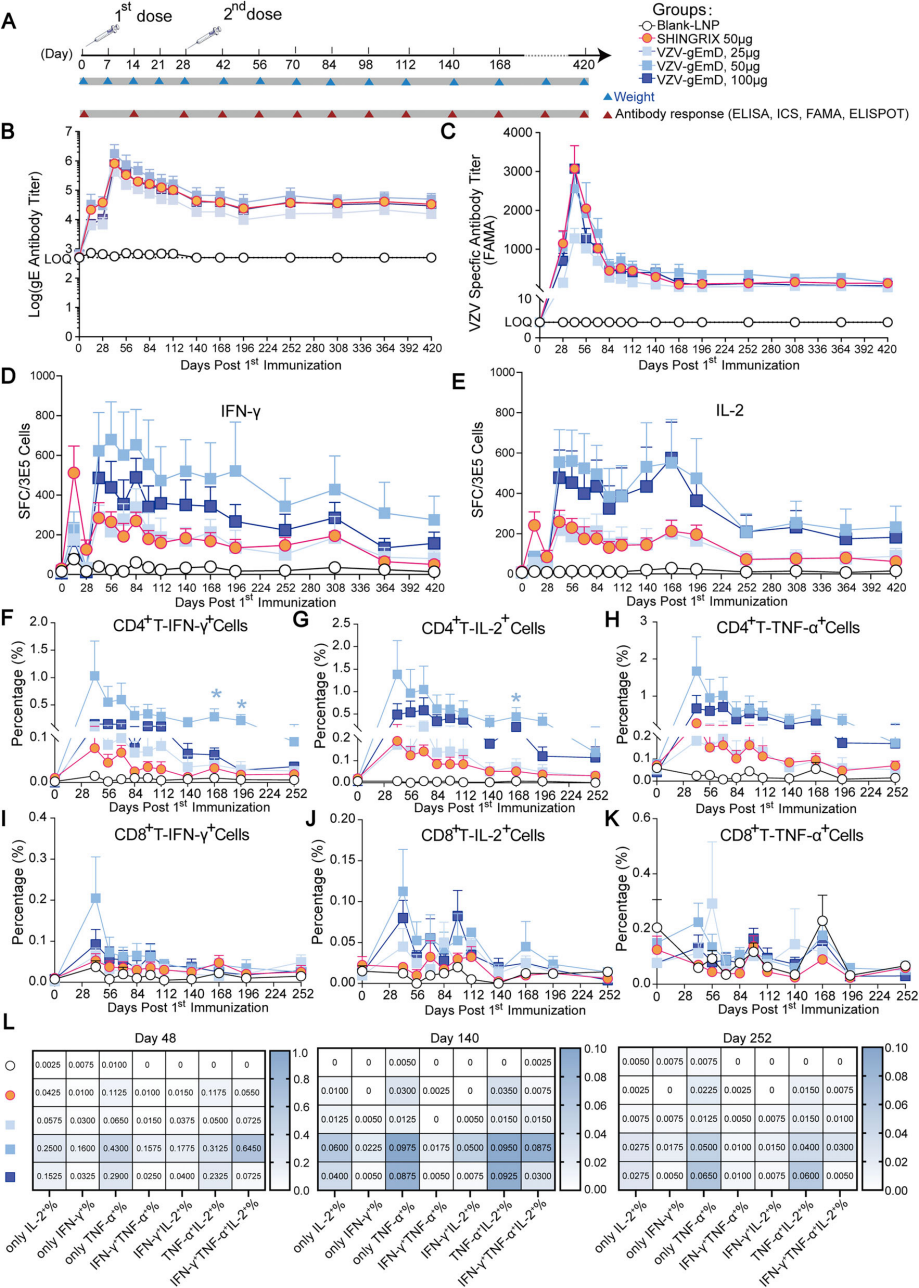
The immunogenicity of VZV‐gEmD injection at various doses in non‐human primates (rhesus macaques). (A) Schematic diagram of the vaccination and sample collection schedule. Rhesus macaques (n=4) were i.m. immunized twice (day 0 and day 28) with blank‐LNP, Shingrix, or VZV‐gEmD at various doses. Blood samples were collected at the indicated time points until day 420. (B) Quantification of gE‐specific IgG antibodies by ELISA. (C) Quantification of VZV‐specific protective antibodies by FAMA. (D) (E) Secreted levels of IFN‐γ and IL‐2 in PBMCs were assessed by ELISPOT assay. (F) (G) (H) Proportions of gE‐specific IFN‐γ‐, TNF‐α‐, and IL‐2‐producing CD4^+^ T cells detected by flow cytometry. (I) (J) (K) Proportions of gE‐specific IFN‐γ‐, TNF‐α‐, and IL‐2‐producing CD8^+^ T cells detected by flow cytometry. (L) Cell frequency of single‐, double‐, and triple‐positive subsets for IFN‐γ, IL‐2, and TNF‐α in response to different VZV‐gEmD doses (25, 50, 100 µg) and controls (Blank‐LNP, Shingrix). Data are represented as mean ± SEM. One‐way ANOVA was used for statistical analysis. *p < 0.05.

Given the significant value of data derived from non‐human primate (NHP) models in understanding human vaccine responses, we conducted a further assessment of the immunogenicity of VZV‐gEmD injection in NHPs. Specifically, we measured gE‐specific IgG antibodies, serum VZV‐specific antibodies, and T‐cell immune responses following two dose intramuscular administration of VZV‐gEmD in rhesus macaques at a 4‐week interval (Figure 9A). For humoral responses, antibody detection was conducted biweekly within the initial 112 days, followed by monthly intervals thereafter. Monitoring has currently reached the day 420. The initial immunization in all VZV‐gEmD and Shingrix groups successfully induced the production of gE‐specific IgG antibodies and VZV‐specific antibodies (Figure 9B,C). The second immunization on day 28 increased the levels of both antibody types and peak antibody levels in all the groups were observed two weeks after the second immunization dose (D42), except for the blank LNP group. Subsequently, antibody levels declined and reached plateau from day 196 onward. The estimated half‐life values are summarized in Figure S10. Longitudinal mixed‐effects analyses demonstrated that the antibody responses persisted for up to 420 days (Figures S8 and S9). For gE‐specific IgG antibodies, the VZV‐gEmD group dosed at 100 µg induced antibody levels comparable to those of the Shingrix group throughout the monitoring period, while the 50 µg VZV‐gEmD group elicited a slightly higher antibody levels than the Shingrix group from day 42 for over one year, although this difference was not statistically significant (Figure 9B). For VZV‐specific antibodies, all three VZV‐gEmD dosage groups induced antibody titers equivalent to Shingrix within the first 168 days. From day 168 onward, the 50 µg group showed persistently, but not significantly, higher titers than Shingrix (Figure 9C). Collectively, these findings highlight the potential of VZV‐gEmD to elicit more potent and durable antibody responses compared with Shingrix in the present experimental setup.

For cellular immune response, it has been reported that VZV‐specific T cell responses play a critical role in the prevention of herpes zoster, particularly the cellular immune responses mediated by Th1‐type CD4⁺ T cells [40, 41]. Therefore, we used both ICS (intracellular cytokine staining) and ELISPOT to detect cytokine secretion and the proportions of relevant T cells. After the first immunization, the secretion of the cytokines IFN‐γ and IL‐2 in the VZV‐gEmD groups was slightly lower than that in the Shingrix group. Secondary booster immunization on day 28 significantly enhanced the cellular immune responses across all groups. Among the three dosage groups of VZV‐gEmD, the 25‐µg low‐dose group exhibited immunological equivalence to Shingrix, whereas the 50‐µg and 100‐µg groups demonstrated superiority, with the 50‐µg group achieving the highest magnitude of enhancement. Two weeks post‐second immunization (D42), the 50 µg VZV‐gEmD group presented over twofold higher levels of IFN‐γ and IL‐2 than did the Shingrix group, yet there was no significant difference. These elevated levels persisted for an extended duration of 420 days (Figure 9D,E; Figures S11 and S12). The 25 and 50 µg groups exhibited a clear dose‐dependent immune response. Notably, the highest dose group (100 µg) showed attenuated immunogenicity. Given existing reports that the production of anti‐PEG antibodies can impair the efficacy of certain mRNA vaccines [42, 43], we conducted corresponding tests to determine whether PEG in the high‐dose group might induce specific antibodies that could affect vaccine performance. However, no anti‐PEG antibodies were detected in rhesus monkeys from any of the vaccine dose groups or the empty LNP group (Table S8). In the current experimental context, proportions of IFN‐γ^+^CD4^+^ cells, TNF‐α^+^CD4^+^ cells, and IL‐2^+^CD4^+^ cells in all VZV‐gEmD groups maintained equivalent to or greater than those in the Shingrix group throughout the monitoring process following the first immunization. Notably, the 50 µg group showed superior cellular immunity to Shingrix at all time points. At two weeks post‐second immunization (D42), these cell proportions in 50 µg group of VZV‐gEmD were 7 to 13 times those of the positive Shingrix group (Figure 9F–H). Moreover, it exhibited significantly higher proportions of IFN‐γ^+^CD4^+^ cells than Shingrix at days 168 and 196 (Figure 9F, *p* < 0.05), and significantly elevated proportions of IL‐2^+^CD4^+^ cells at day 168 (Figure 9G, *p* < 0.05). Until day 196, proportions of IFN‐γ^+^CD4^+^ cells, TNF‐α^+^CD4^+^ cells, and IL‐2^+^CD4^+^ cells in VZV‐gEmD 50 µg group remained sevenfold to tenfold higher than those in the Shingrix group (Figure 9F–H), indicating sustained immunogenicity over time. Consistent with the findings obtained in mice, there was no observable difference in the quantity of cytokine‐positive CD8^+^ T cells among the different groups (Figure 9I–K). While in the NHP models, the pattern of functional responses differed slightly from that observed in the mouse model. Single expression of IFN‐γ, IL‐2, or their combined expression was primarily observed in the Shingrix group. The VZV‐gEmD groups again demonstrated superiority in secreting combinations of two or three cytokines. Despite a gradual decline over time, the cytokine secretion levels remained superior to those of Shingrix (Figure 9L).

During the experiments, body weight of NHPs was found to be constant for both Shingrix and our mRNA‐LNP vaccines (Figure S13). No abnormalities in animal behavior, coordination of the upper and lower limbs, physical appearance, mental state, or responses to external stimuli were observed for all the animals. These results suggested that the mRNA‐LNP vaccines demonstrated a favorable safety profile in NHPs.

Therefore, VZV‐gEmD injection was able to elicit robust humoral and cellular response in NHPs, along with high safety profiles and long‐lasting immunity persisting for nearly one year.

### 2.9 In Vivo Biocompatibility and Safety of VZV‐gEmD Injection in SD Rats

**FIGURE 10:**
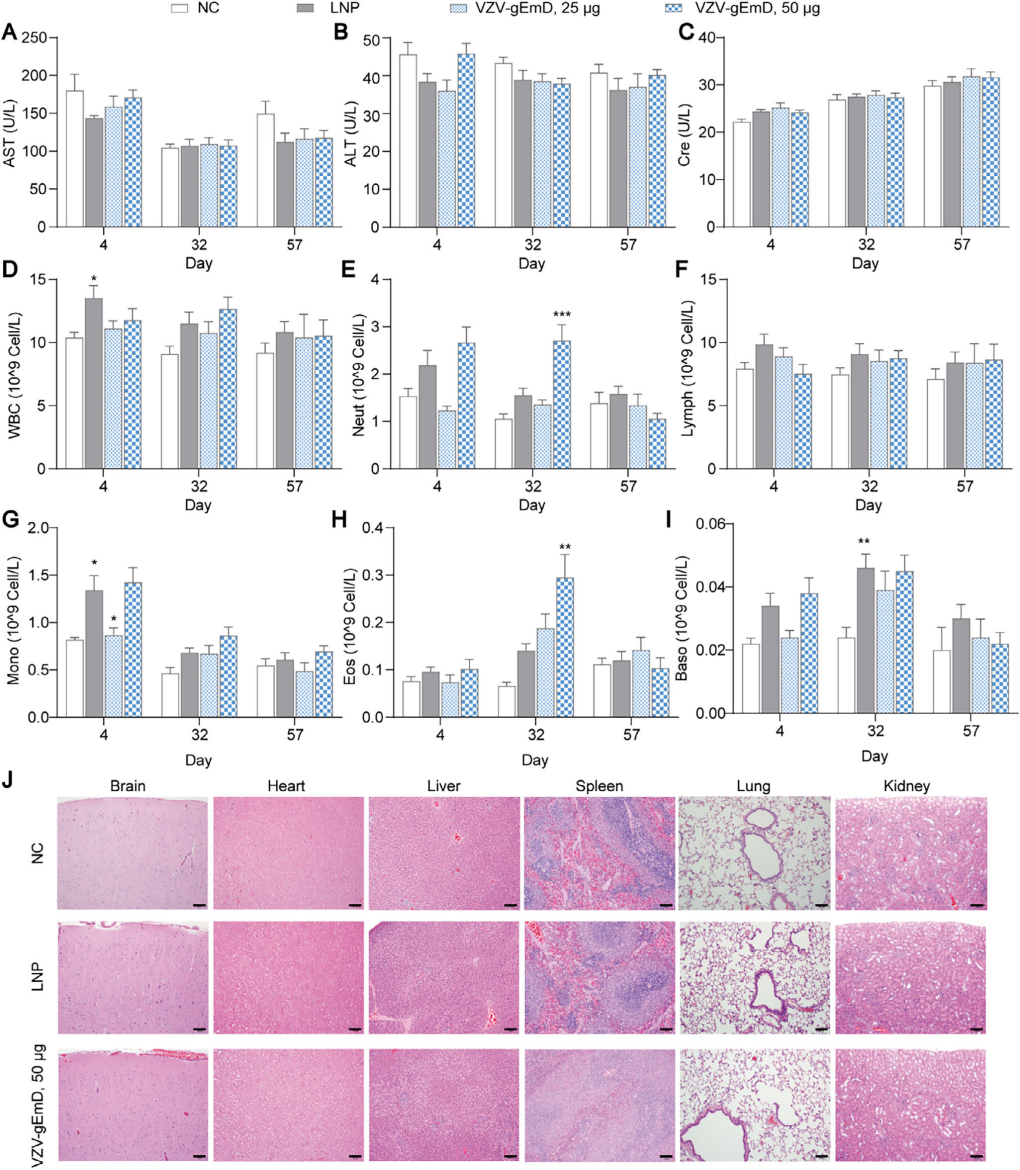
Safety evaluation of SD rats within 57 days after administration of negative control (CN), LNP, and VZV‐gEmD injections. Changes in (A) aspartate aminotransferase (AST), (B) alanine aminotransferase (ALT), (C) creatinine (CRE), (D) white blood cell count (WBC), (E) neutrophils (Neut), (F) lymphocytes (Lymph), (G) monocytes (Mono), (H) eosinophils (Eos), and (I) basophils (Baso). (J) H&E staining of main organs. Data are represented as mean ± SEM. One‐way ANOVA was used for statistical analysis. *p < 0.05, **p < 0.01.

The biocompatibility of the VZV‐gEmD injection was evaluated to assess its safety in SD rats. The animals were immunized once every two weeks over a four‐week period, for a total of three injections. For biochemical analysis, no significant differences were observed in the levels of aspartate aminotransferase (AST), alanine aminotransferase (ALT), and creatinine (Cre) between the treatment groups on day 4, 32 and 57, indicating that the VZV‐gEmD injection had no hepatotoxicity and nephrotoxicity effects (Figure 10A–C). The hematological analysis includes measurements of white blood cells (WBC), neutrophils (Neut), lymphocytes (Lymph), monocytes (Mono), eosinophils (Eos), and basophils (Baso). Specifically, the LNP group exhibited a modest increase in WBC and Mono counts during the administration phase (Day 4), accompanied by a rise in Baso counts at the end of the administration period (Day 32). For the VZV‐gEmD injection, the low dose induced a mild increase in Mono counts during the administration phase (Day 4), whereas the high dose caused an elevation in Neut and Eos counts at the conclusion of the administration period (Day 32). However, these subtle hematological changes returned to levels comparable to those of the NC group during the post‐administration recovery phase (Day 57) (Figure 10D–I). Additionally, microscopic observations were conducted on pathological changes in the administration site and main organs. At the end of dosing (Day 32), slight to mild intramuscular/perimuscular inflammatory cell infiltration, edema, necrosis, fibrosis, hemorrhage, neovascularization, and muscle fiber degeneration/necrosis were observed at the administration site in the LNP group and VZV‐gEmD groups (Table S9). H&E staining of major organs revealed no obvious damage or pathological changes in the brain, heart, lungs, and kidneys across all treatment groups, except for mild hepatocellular vacuolation and splenic lymphocytosis observed in the VZV‐gEmD Group (Figure 10J). Following the recovery period (Day 57), lesions at the administration site tended to resolve, while pathological changes in the liver and spleen were completely reversed (Table S10), indicating no apparent biological toxicity in vivo. These findings afforded great confidence for further clinical evaluation of our vaccine candidate.

## 3 Discussion

Currently, Shingrix dominates the market of VZV vaccines, benefiting from its high and enduring protective ability regardless of age. However, the high reactogenicity and high cost of Shingrix demonstrated an urgent need for novel vaccines with both superior immunogenicity and safety. In view of the critical role of CMI in HZ prevention, mRNA vaccines become an ideal option due to its potency in inducing cellular immunity. Here, we developed a novel mRNA‐LNP vaccine based on VZV glycoprotein E to prevent HZ. We obtained a series of mRNA‐LNP candidate vaccines with the assistance of AI technology and optimized several 5’UTR elements by assessing their immunogenicity in mice. A 4‐week administration interval for the candidate vaccine resulted in the most potent immunogenicity. The mRNA‐LNP vaccine VZV‐gEmD successfully stimulated a strong humoral response, including gE‐specific IgG antibodies and VZV‐specific antibodies, in NHPs. Notably, CD4^+^ T‐cell responses were significantly stronger and longer‐lasting in the VZV‐gEmD group than in the Shingrix group. Moreover, the relationship between antigen localization in cells and immunogenicity was investigated. Our mRNA‐LNP‐based vaccine has potential for clinical development as an effective prophylactic vaccine against HZ.

To generate more potent mRNA vaccines than recombinant vaccines, we designed a series of mRNA variants based on gE antigen mutants and screened by AI technology. This advanced technology exhibited superior screening efficiency of candidate vaccines, surpassing traditional vaccine development methods. Notably, our current AI model functions as a supplementary screening tool developed through training on empirically derived sequences. Although it does not possess the capability to autonomously design VZV vaccines, the model exhibits strong applicability and provides significant insights to inform the rational design of VZV vaccines in future research. The comparative analysis indicated that compared with recombinant vaccines, all the mRNA‐LNP vaccines elicited superior gE‐specific IgG antibody responses and CD4^+^ T‐cell responses in mice (Figure 3). This finding was aligned with previous studies. mRNA vaccines encoding full‐length gE antigens or their truncated variants were reported to induce superior immunogenicity and lower immunoreactivity than Shingrix [23, 24]. Moreover, a novel mRNA‐LNP vaccine for HZ prevention, developed by Moderna recently, was found to induce both humoral and cellular immune responses that were comparable to those induced by Shingrix in rhesus macaque models [22]. A VZV mRNA vaccine candidate induced superior cellular immunity and comparable humoral and Fc‐mediated immunity compared to the licensed subunit vaccine in a mouse model [25]. All these results demonstrated the superior immunogenicity of mRNA vaccines than traditional vaccines.

The underlying correlation between immunogenicity and gE localization has drawn extensive attention, but not been fully understood [30, 31, 34, 35]. We performed a series of site mutations targeting key functional domains of the gE protein to modify antigens localization, hypothesizing these changes would enhance processing and presentation of antigens expressed by the mRNA‐LNP vaccine [31, 34], and thereby promote both humoral and cellular immune responses. Our results showed that the full length antigen encoded by VZV‐gEmD, exhibiting greater cell membrane accumulation and reduced TGN retention, elicited higher immunogenicity than the other truncated sequences. These findings align with a recent comparable study [26], which revealed that gE proteins with dual localization at the cell membrane and Golgi apparatus demonstrated the most potent immunogenicity, followed by membrane‐predominant localization, while Golgi‐retained gE was least immunogenic. Collectively, these observations support the notion that surface expressed gE tends to elicit a more robust immunogenic response. We proposed potential mechanisms based on current knowledge. On one hand, the Golgi apparatus serves as a critical site for protein glycosylation modification [44]. In a study on chimeric antigen receptor T‐cell (CAR‐T) therapy, researchers demonstrated that hyperglycosylation of antigens mediated by the Golgi apparatus significantly impairs antigen‐antibody binding [45]. If gE is excessively retained in the Golgi apparatus, it is highly likely that hyperglycosylation would mask neutralizing epitopes, thereby compromising the immune response; on the other hand, membrane‐localized antigen simultaneously activate two pathways: direct recognition by B cell receptors (BCRs) initiating B cell activation, and internalization by professional antigen‐presenting cells (APCs) for MHC class II presentation to CD4^+^ T cells stimulating cytokine secretion that amplifies the CD4^+^ T cell response but also further support B cell differentiation and the production of virus‐neutralizing antibodies [46]. In contrast, intracellularly retained antigens primarily activate a single immune pathway: proteasomal processing into small peptide fragments for MHC class I presentation to CD8^+^ cells, triggering a cellular immune response [47]. Therefore, membrane‐localized antigen ultimately induces a stronger overall immune response compared to intracellularly retained antigen. Clarification of the specific mechanisms requires further validation through approaches such as glycosylation mass spectrometry analysis, so as to provide more definitive guidance for the sequence design of VZV vaccine development.

Subsequently, the immunogenicity elicited by the resulting vaccine candidate was comprehensively assessed. As reported, the decline in the VZV‐specific cellular immune response served as a factor for an increased frequency and severity of HZ [48], while the consistency of post‐varicella infection antibody responses are insufficient to inhibit the induction of HZ [49, 50]. This observation may be attributed to the pathogenesis of HZ. As documented in the literature, during the development of HZ, VZV disseminates to the cutaneous epithelium via nerve fibers and primarily persists in an intracellular manner, accompanied by low‐level viremia in the bloodstream [25, 51]. Consequently, neutralizing antibodies exhibit limited efficacy in eliminating reactivated VZV. In contrast, the early phase of HZ is characterized by the predominant infiltration of neutrophils, macrophages, and natural killer cells into the affected cutaneous lesions, with T cell infiltration occurring subsequently in the later phase [2]. Therefore, VZV‐specific antibody responses are commonly considered not essential for the prevention of HZ. However, the majority of researchers have utilized this immune response to evaluate both the effectiveness and longevity of developed vaccines [11, 22, 23, 24]. In our work, we monitored the gE‐specific IgG antibodies by ELISA and the protective VZV‐specific antibodies by FAMA. Compared with Shingrix, the humoral immunity elicited by our mRNA vaccine was sustained at a higher level for an extended duration of over one year, suggesting that the mRNA vaccine was capable of generating a strong and enduring immune response. Typical neutralization assays were not incorporated in the present study, despite its potential to facilitate a more comprehensive assessment of humoral immunity. Instead, the primary focus of our investigation was placed on the evaluation of cellular immunity.

T‐cell‐mediated immunity, particularly involving antigen‐specific CD4^+^ T cells, is critical for protecting the host from reactivation of latent VZV [32, 33]. In clinical studies, higher levels of VZV‐specific T cells were significantly negatively correlated with the risk of herpes zoster incidence and disease severity. Meanwhile, the gE‐specific CD4⁺ Th1 cell responses induced by the licensed positive control vaccine Shingrix showed a significant positive correlation with clinical protective efficacy [52, 53]. These studies collectively confirm the critical role of CD4⁺ T cells in mediating protective immunity induced by VZV vaccines. Therefore, in the efficacy evaluation of novel VZV candidate vaccines, the induction of potent VZV‐specific CD4⁺ T cell responses will serve as a key indicator for assessing vaccine efficacy. In both the mice and rhesus macaque models, our mRNA‐LNP vaccines elicited significantly elevated levels of IL‐2 and INF‐γ secretion, as well as an increased proportion of cytokine‐positive CD4^+^ T cells, in comparison with those elicited by the Shingrix vaccine. The outstanding performance of our mRNA‐LNP vaccine could be attributed to its unique mechanism of intracellular antigen translation, which results in a high fidelity of posttranslational modifications and efficient induction of CD4^+^ T‐cell responses via MHC class II presentation [22, 54]. However, VZV‐specific CD8^+^ T‐cell responses were not observed, which aligns with findings from prior studies [22, 55, 56]. This phenomenon may result from a limited number of CD8^+^ T‐cell epitopes on the gE (ORF68) antigen. Because VZV‐specific CD8^+^ T cells can predominantly exhibit reactivity toward ORF9 rather than other VZV antigens, such as gE and gB [57]. The current study has not examined the relationship between the lack of CD8⁺ T‐cell responses and critical efficacy outcomes to establish a functional correlation. Incorporating such analyses would substantially enhance the comprehensive interpretation of the findings reported herein. The durability of vaccines is also an important factor affecting the effectiveness of vaccine protection [58]. The CD4^+^ T‐cell immunity elicited by our vaccine was sustained at an elevated level for an extended duration. Compared with the reported prolonged protection for a period of 85 days [11], immunogenicity of our vaccine was shown to persist for 420 days (Figure 9).

Moreover, the 50 µg VZV‐gEmD group exhibited the strongest immune responses, while higher doses up to 100 µg did not enhance immunity. Our current data demonstrate that the decrease in vaccine efficacy is not associated with anti‐PEG antibody production. Given the small sample size (n = 4 per group), a detailed analysis of immunogenicity and immune persistence data revealed one animal in the VZV‐gEmD high‐dose group (100 µg/animal) with weaker gE‐specific IgG and VZV‐specific antibody levels, and a faster decline over time than other group members. This led to the high‐dose group's immune response trending lower than the medium‐dose group (50 µg/animal). For cellular immunity, this animal exhibited significantly fewer IFN‐γ and IL‐2 positive spots from 14 days post the second immunization (D42), with a marked decrease at D196, while other high‐dose group animals maintained high spot counts. These data indicate substantial individual differences in immunogenicity and persistence post VZV‐gEmD immunization, despite no abnormalities in general conditions, body weight, temperature, or food intake over the 420‐day study. In summary, the small sample size and individual variability could be the main reasons for the medium‐dose group's slightly higher immunogenicity than the high‐dose group at certain time points.

In addition to potent and durable immunogenicity, the safety profile of VZV‐gEmD was consistent with the established safety characteristics of LNP‐mRNA vaccines. The biochemical and pathological parameters exhibited by the current vaccine are consistent with the common manifestations of LNP‐mRNA vaccines, with analogous observations well‐documented in COVID‐19 mRNA vaccines (BNT162b2/mRNA‐1273). Moreover, these phenomena occur transiently during the post‐administration period and are completely reversed during the recovery phase subsequent to dosing. Overall, the VZV‐gEmD vaccine exhibited greater immunogenicity, more durable immunity, and better safety than the licensed Shingrix vaccine. These findings position VZV‐gEmD as a promising candidate for the prevention of VZV infection.

There are also limitations of our study that remain to be further addressed. First, in the UTR optimization process, a comparative screening approach was utilized to identify the optimal UTR sequence, leveraging natural or rationally modified UTRs derived from highly expressed human genes. This strategy was designed to align with the demand for expedited vaccine development, prioritizing efficiency while ensuring biological relevance. However, investigating the potential regulatory mechanisms underlying these high‐expression UTRs holds significant importance. It not only clarifies the rationale behind the optimization strategy but also provides a more explicit reference framework for future UTR‐based optimization endeavors in vaccine development. Second, the safety evaluation in rhesus monkey models was limited to monitoring body weight and clinical observations. More comprehensive assessments of the safety profile were not conducted due to restricted access to sample resources. Therefore, SD rats were used as an alternative model, but cross‐species extrapolation of safety data may have limitations.

To summarize, this study proposes a novel strategy for developing an mRNA‐LNP vaccine that provides effective and long‐lasting immunity against VZV infection. First, we developed several candidate sequences and screened them using an AI‐assisted pipeline to identify the most promising one. Considering that the UTR is also essential for protein replication and antigen translation efficiency, we optimized the UTR incorporated into our vaccine design. Second, we investigated the immunization intervals and dosages of the vaccine. The developed platform demonstrated a substantial capacity to elicit potent and long‐lasting humoral and cellular immune responses in mice and rhesus macaques, providing reference data for human vaccine development. The current work demonstrated the successfully utilization of AI technology in the development of VZV vaccines, established an advanced standard system for vaccine evaluation, and promoted the application of mRNA vaccines for HZ prevention.

## 4 Materials and Methods

### 4.1 Materials

Dimethyl sulfoxide (DMSO) and ionomycin (ION) was purchased from Sigma‐Aldrich (Shanghai, China). Dulbecco's modified Eagle's medium (DMEM), RPMI Medium 1640, fetal bovine serum (FBS), Trypsin, penicillin and streptomycin, and L‐Glutamine were obtained from Gibco Co., Ltd (Grand Island, USA; Shanghai/Suzhou, China). RIPA lysis buffer, 10×RBC lysis buffer, and OPTI‐MEM were obtained from Thermo Fisher Scientific (Shanghai, China). 10×PBS and PMSF were purchased from Beyotime Biotechnology Co. Ltd (Shanghai, China). Varicella Zoster Virus Glycoprotein E (VZV gE) ELISA Kit (For Vaccine Development) was obtained from ACROBiosystems (Beijing, China). Anti‐VZV gE antibody, Goat anti‐mouse IgG H&L, and rabbit anti‐mouse IgG H&L were purchased from Abcam Trading Co. Ltd (Shanghai, China). CoraLite Plus 647‐conjugated GOLGA2/GM130 polyclonal antibody and CoraLite594‐conjugated TGN46 polyclonal antibody were purchased from proteintech Group, Inc (Wuhan, China). Donkey anti‐mouse immunoglobulin G (IgG) conjugated to Alexa Fluor 488 was obtained from Yeason Biotechnology Co., Ltd (Shanghai, China). Recombinant VZV (strain Oka vaccine) Envelope Glycoprotein E was purchased from Novoprotein Scientific Inc (Suzhou, China). Tween‐20 and 10×PBST were obtained from Solarbio Science Technology Co., Ltd (Beijing, China). ELISPOT assay kits (IL‐2, IFN‐γ, TNF‐α) for both mouse and monkey were purchased from Mabtech (Nacka Strand, Stockholm County, Sweden). Fluorescent anti‐mouse/human antibodies for CD4, CD8, IL‐2, IFN‐γ, and TNF‐α were all purchased from Biolegend, Inc (San Diego, California, USA). Antibodies for CXCR5, OX40, CD137, CD19, Fas, GL7, IgD, IgM, and His were also purchased from Biolegend, Inc (San Diego, California, USA). Isoflurane was purchased from RWD Life Science (Shenzhen, China). Human embryonic kidney (HEK) 293T cells were purchased from Nanjing KeBai Biotechnology Co., Ltd (Nanjing, China). MeWo cells were purchased from Beyotime Biotechnology Co. Ltd (C6573, Shanghai, China).

### 4.2 Development of the Reference Difference Transformer (RDTransformer)

#### 4.2.1 Datasets

A total of 60,005 RNA sequences were retrieved from the RNAcentral database V18 [59]. The dataset was subjected to quality filtering to remove duplicates, sequences containing non‐canonical ribonucleotides, and sequences outside the 50–2,048 nt range. Redundant sequences were subsequently removed using CD‐HIT‐EST (v4.8.1) [60] at an 85% identity threshold, yielding 38,712 non‐redundant representatives with the following class distribution: lncRNA (7,910), miRNA (3,533), rRNA (4,478), snRNA (7,944), snoRNA (7,727), and tRNA (7,120). The pre‐training dataset was partitioned into training (90%) and validation (10%) subsets using stratified random sampling to maintain proportional representation of each RNA type.

The fine‐tuning dataset comprised of 514 unique mRNA sequences encoding VZV gE variants. Protein expression levels and immunogenicity for each variant were measured by Western blot (WB) and ELISA, respectively. Continuous measurements were binarized using predetermined thresholds (1 for WB, 500 for ELISA), assigning two binary labels to each sequence, which resulted in distributions of WB (266+/248−) and ELISA (267+/247−). For each fine‐tuning task, the dataset was stratified by its respective labels and split into training and independent hold‐out test sets at an 80:20 ratio. The training set was used for 4‐fold cross‐validation to optimize hyperparameters and assess stability, while the test set was reserved for final evaluation.

To compute position‐wise difference embeddings for rationally designed mutant sequences, the wild‐type VZV gE mRNA sequence (Sequence ID: QXN54923.1) was used as a reference. All variant sequences were aligned to the wild‐type sequence using MAFFT (v7.525, Auto mode) [61]. Alignment gaps were represented by “−” to preserve position‐wise correspondence for the token‐level difference embeddings.

#### 4.2.2 Sequence Tokenization and Preprocessing

The variant and wild‐type VZV E mRNA sequences were tokenized using overlapping 3‐mer tokens, generated by a sliding window of size 3 and stride 1. This process defined a fixed vocabulary of 64 trinucleotides, each mapped to a unique integer index from 1 to 64, with 0 reserved for padding. To ensure uniform input dimensions, all sequences were zero‐padded to a fixed length of 2048, chosen to exceed the maximum sequence length in the dataset. Since the pre‐trained model was built from unaligned sequences, its vocabulary contained no alignment token. Therefore, during fine‐tuning, any 3‐mer containing an alignment gap was mapped to the padding index to maintain compatibility. Correspondingly, a binary attention mask was created for each sequence to prevent attention to padding positions.

#### 4.2.3 Architecture of the RDTransformer

The proposed model— Reference Difference Transformer (RDTransformer) was built upon a Transformer encoder architecture [62]. Input tokens were embedded into a 64‐dimentional vectors using an embedding layer with a vocabulary size of 65. Sinusoidal positional encodings of the same dimensionality were added to retain sequence order information. The encoder consisted of 2 layers, each with an embedding dimension of 64, 4 attention heads, and a feed‐forward network with a hidden dimension of 192.

To isolate representational changes attributable specifically to introduced mutations, a Difference Extraction Module was incorporated. For each mini‐batch, the wild‐type reference sequence was encoded using the current encoder parameters to ensure representational consistency with the variant sequences. Token‐wise difference embeddings were computed by subtracting the reference embedding from the variant embedding, and positions with identical tokens were further explicitly masked to zero to remove background signals from non‐mutational sites. Masked max‐pooling over non‐padding positions then aggregated the most salient mutation‐related differences into a fixed‐length vector that was passed to the downstream classifier, thereby highlighting mutation‐driven changes in the learned representations.

The model was first pre‐trained on a large ncRNA corpus using a six‐class RNA categorization task to learn generalizable representations of RNA sequences. For the downstream task, the model was fine‐tuned with the following adaptations: (a) the pre‐trained token embedding layer and Transformer encoder were fine‐tuned; (b) the Difference Extraction Module was incorporated to capture mutation‐specific representational differences; and (c) the six‐class linear classification head was replaced by a trainable binary classifier. This new classifier was initialized using Xavier uniform initialization [63], and incorporates a residual connection with layer normalization to stabilize gradient propagation. Complete model specifications are provided in Table S11.

#### 4.2.4 Model Training

The Transformer encoder was pre‐trained using the AdamW optimizer with weight‐decay. A combined learning‐rate schedule was applied, consisting of a linear warmup followed by cosine‐annealing decay. Gradient‐norm clipping was used to stabilize training. Early stopping was employed based on validation‐loss progression, and the checkpoint with the lowest validation loss was retained for downstream fine‐tuning. To mitigate class imbalance, an inverse frequency‐weighted cross‐entropy loss was used.

The fine‐tuning model was initialized from the optimal pre‐trained checkpoint and fine‐tuned using the same optimization scheme. For cross‐validation, each fold was trained for a fixed number of epochs (65 for protein‐expression prediction and 52 for IgG‐level prediction), and the best model in each fold was selected based on peak validation AUROC. The median of these optimal epochs (64 and 52) was then adopted as the training length for the final model trained on the full training set. This final model was evaluated on the independent hold‐out test set. Because the fine‐tuning dataset was class‐balanced, standard cross‐entropy loss was used without class weighting. Full training hyperparameters are listed in Table S12.

All experiments were carried out on an Alibaba Cloud Linux 3 server equipped with an NVIDIA A100 GPU. The computational environment consisted of Python 3.11.7, PyTorch 2.6.0 (CUDA 12.4), and scikit‐learn 1.4.0, and a fixed random seed was applied throughout to ensure reproducibility.

#### 4.2.5 Model Evaluation

The pre‐trained model was assessed on the validation set using macro‐averaged AUROC, weighted and macro F1 scores, and confusion matrices. The discriminative performance of the fine‐tuned model was evaluated using AUROC and AUPRC. Calibration was assessed using calibration curves and the Brier score. For 4‐fold cross‐validation, the mean and standard deviation (SD) of these metrics were calculated across the training folds to assess model stability. Additionally, out‐of‐fold predictions were pooled to compute overall cross‐validated performance metrics. Metric uncertainty was estimated via bootstrap resampling with 500 iterations and reported as 95% confidence intervals (95% CI) for both cross‐validation and test‐set evaluation.

#### 4.2.6 Ablation Study

The contribution of key components of the RDTransformer model was evaluated by an ablation study using 4‐fold cross‐validation on the training set, as described in the Model Evaluation section. Specifically, the following variants were compared: (a) the model without pre‐training; (b) the model without the reference sequence input; (c) the model without position‐wise same‐token masking; (d) the model without positional encoding; (e) the model using mean pooling instead of max pooling over padding‐masked sequences.

### 4.3 Preparation of mRNA‐LNP Vaccines

The VZV mRNA vaccine was prepared as previously reported with slight modifications [64]. In brief, the immunogen encoding the VZV gE protein was synthesized in vitro via T7 polymerase‐mediated transcription utilizing a linearized DNA template. The resulting methyl‐pseudouridine‐modified mRNAs were subsequently capped with the Cap1 analogue reagent and purified using Monarch RNA purification columns (New England Biolabs, Ipswich, Massachusetts, USA). Following purification, the mRNAs were resuspended in TE buffer to achieve the specified concentration.

For the encapsulation of mRNAs, lipid components (YK‐009: 1,2‐distearoyl‐sn‐glycero‐3‐Phosphocholine (DSPC): Cholesterol: 1,2‐dimyristoyl‐rac‐glycero‐3‐methoxypolyethylene glycol 2000 (DMG‐PEG2000) = 49: 10: 39.5: 1.5, mol%) were solubilized in ethanol and subsequently combined with mRNAs. This process was conducted using microfluidic technology, and the formulations were diluted with PBS and subsequently subjected to ultrafiltration. The vaccine formulations were then assessed comprehensively.

### 4.4 Protein Expression Assay

Human embryonic kidney (HEK) 293T cells were cultured in high‐glucose Dulbecco's modified Eagle's medium supplemented with 10% fetal bovine serum (FBS) and 1% penicillin‒streptomycin. gE‐mRNAs were transfected into the cells, and the cells were then incubated for 18 h to allow sufficient time for mRNA translation and protein expression. Subsequently, the cell lysates were prepared in RIPA lysis buffer and the protein samples were collected after centrifugation (4°C, 12000 rpm, 10 min). The expression levels of gE protein were analyzed based on the protocol of the Varicella Zoster Virus Glycoprotein E (VZV gE) ELISA assay kit (ACROBiosystems).

### 4.5 Localization of Candidate Antigens Observed via Laser Scanning Confocal Microscopy

MeWo cells were seeded in confocal dishes at 37°C in a cell incubator and incubated overnight. The cells were subsequently transfected with various agents, specifically VZV‐gEmD, VZV‐gEmP, VZV‐gEmA, and VZV‐gEmQ. After 48 h, the cells were fixed, permeabilized, and blocked for 2 h at ambient temperature. The cells were subsequently incubated overnight at 4°C with an anti‐VZV gE antibody, a CoraLite Plus 647‐conjugated GOLGA2/GM130 polyclonal antibody (proteintech), and a CoraLite594‐conjugated TGN46 polyclonal antibody (proteintech). After washing with PBS, the samples were stained with a secondary antibody, donkey anti‐mouse immunoglobulin G (IgG) conjugated to Alexa Fluor 488 (Yeasen). The cells were subsequently washed with PBS. DAPI solution was used to stain the cell nuclei. A confocal laser scanning microscope was used for image acquisition.

### 4.6 Ethics, Animals, and Immunization

All the animal experiments were conducted in compliance with the guidelines for the care and use of laboratory animals, and all experiments involving laboratory animals were approved by the Institutional Animal Care and Use Committees (IACUCs) of JOINN Laboratories (Beijing: No. B‐ACU24‐0281; Suzhou: S‐ACU24‐0764) and Wuxi Apptec (No. PD13‐CD059‐2024v1.0).

For the mouse study, six‐week‐old female specific pathogen‐free (SPF) BALB/c mice (15–18 g) were acquired from Vital River Laboratory Animal Technology Ltd. (Beijing, China). The mice were randomly assigned to groups consisting of six to eight individuals each (n = 6∼8) and were maintained under SPF conditions. They were housed at Central Animal Services of the Institute of Medical Biology, Chinese Academy of Medical Sciences (IMB, CAMS), with unrestricted access to food and water. The Shingrix group was used as a positive control and dosed at 5 µg per mouse aligned with previous reports [11, 23, 24]. The blank LNP group was used as a negative control. The mice were immunized intramuscularly (i.m.) in the thigh muscle twice with 2‐10 µg candidate vaccines. At predetermined time points, blood samples were collected from submandibular vein or the orbital venous plexus under isoflurane anesthesia. All mice were euthanized via cervical dislocation at terminal time points and the spleens were obtained for further analysis.

For the rhesus macaque study, the animals were individually housed in stainless‐steel cages, which were regularly cleaned. The temperature was set at 18°C–26°C, and the relative humidity was maintained at 40%–70%. The air was exchanged at least 10 times per hour. A time‐controlled lighting system was adopted, providing a regular 12‐h light/12‐h dark circadian cycle from 7:00 a.m. to 7:00 p.m. The rhesus macaques were randomly divided into 5 groups (n = 4, half male and half female) and intramuscularly immunized twice at a 4‐week interval. The dosages of VZV‐gEmD were set at 25, 50, and 100 µg. The Shingrix served as positive control and dosed at 50 µg [11, 22]. The blank LNP group was set as negative control. At the predetermined time points, with the rhesus monkeys properly restrained while in a non‐anesthetized and conscious state, blood was collected from the saphenous vein or cephalic vein. All blood samples were placed on ice after collection and centrifuged (3000 g, 4°C, 10 min) within 1 h. The separated plasma and serum were stored in a ‐80°C freezer for future use. The remaining blood cells were immediately used for the isolation of peripheral blood mononuclear cells (PBMCs). The PBMCs were freshly prepared and used immediately. Body weight was monitored longitudinally to analyze safety profiles and no euthanasia was performed on the rhesus monkeys.

For the SD rat study, six to seven‐week‐old male/female specific pathogen‐free (SPF) SD rats were acquired from Vital River Laboratory Animal Technology Ltd. (Zhejiang, China). The rats were randomly divided into 4 groups (n = 10): a negative control group (NC), a Blank‐LNP group, a low dose VZV‐gEmD group (25 µg) and a high dose VZV‐gEmD group (50 µg). The rats received intramuscular injections once every two weeks for a period of 4 weeks, totaling 3 doses. Throughout the experiment, clinical observations, including assessments of the local injection site, were conducted. During the treatment phase (Day 4), jugular venous blood samples were harvested for complete blood count (CBC) and clinical biochemistry profiling. At the termination of the treatment phase (Day 32) and the recovery phase (Day 57), prior to scheduled euthanasia, animals were anesthetized via isoflurane inhalation, followed by abdominal aortic blood collection for the quantitative assessment of hematological indices and clinical biochemistry parameters. The animals underwent gross anatomical examination, and the brain, heart, liver, spleen, lung, and kidneys were harvested for pathological analysis via hematoxylin and eosin (HE) staining.

### 4.7 Assessment of gE‐Specific IgG Antibodies

Following the clotting of whole blood at 4°C overnight, immunized mouse sera were obtained via centrifugation at 3000 g for 10 min. The gE protein (0.5 µg/mL) was used to pre‐coat 96‐well plates via incubation at 4°C overnight. The plates were subsequently blocked with 1% bovine serum albumin (BSA) at 37°C for 2 h, after which they were incubated with two serial dilutions of mouse serum at 37°C for 1 h. Bound antibodies were identified by using a rabbit anti‐mouse IgG‐horseradish peroxidase (HRP) conjugate as the secondary antibody (Abcam). The plates were subsequently developed with tetramethylbenzidine (TMB) as the substrate, and the reaction was terminated by the addition of 2 mol/L sulfuric acid. The absorbance was measured at 450 nm using a spectrophotometer (Thermo, Shanghai, China) and the antibody titers were calculated accordingly. The levels of gE‐specific IgG antibodies in rhesus monkey plasma samples were determined using the same method.

### 4.8 Measurement of gE‐Specific IgG Subclasses

Th1‐dependent IgG2a and Th2‐dependent IgG1 antibody subclasses were measured by ELISA using sera from vaccinated mice. Briefly, gE protein was coated onto 96‐well plates at a concentration of 1 µg/mL and incubated overnight at 4°C. The plates were washed three times with PBS containing 0.05% Tween‐20 (PBST) and blocked for 2 h at ambient temperature. Serially diluted serum samples were added and incubated for 1 h at 37°C, followed by the addition of HRP‐conjugated goat anti‐mouse IgG1 or IgG2a antibodies (Abcam). After a 1‐h incubation at 37°C, TMB substrate was added for color development, and absorbance was measured at 450 nm. Data were collected and analyzed using Gen5 CHS Software (Version 3.11).

### 4.9 Fluorescent‐Antibody‐to‐Membrane‐Antigen (FAMA) Test

The VZV‐specific antibodies in rhesus monkey serum samples were detected using the FAMA method. MRC‐5 cells were infected with VZV‐Ellen. After observing that 80%–90% of the monolayer cells showed cytopathic effects, the cells were digested with 0.25% trypsin at 37°C for 2 min, and then the trypsin was discarded. Cell culture medium was added to gently dislodge the diseased cells. After centrifugation (400 g, 5 min), the cells were collected and resuspended in Dulbecco's Phosphate‐Buffered Saline (DPBS). Subsequently, glass slides bearing infected cells were prepared from the resulting cell suspension and stored for further use. Serum samples of rhesus monkey were subjected to heat inactivation in a 56°C water bath for 30 min and subsequently added onto the prepared glass slides. The slides were then incubated in a humidified chamber overnight incubated with FITC‐conjugated secondary antibody for 30 min. After mounting the slides, microscopic examination was performed.

### 4.10 Enzyme‐Linked Immunospot (ELISPOT) Assay

Dispersions of mouse spleens were obtained using a 70 µm cell strainer. Following the lysis of red blood cells with RBC lysis buffer at room temperature for 5 min, the splenocytes were re‐suspended in serum‐free medium at a final concentration of 3 × 10^5^ cells per well in a 96‐well plate. Subsequently, the peptide pool was introduced at a final concentration of 1 µg/mL. An overlapping peptide library targeting the full length gE protein was used here and each peptide is 15 amino acids in length with an 8‐amino acid overlap between adjacent peptides. The mixture was incubated overnight. The frequencies of T cells that secreted IFN‐γ, IL‐2, or TNF‐α were evaluated utilizing commercially available ELISPOT assay kits (Mabtech) following the manufacturer's instructions. The spots were developed using BCIP/NBT substrate and subsequently quantified with a ELISPOT Analyzer (AID, Freiburg, Germany). Samples of peripheral blood mononuclear cells (PBMCs) collected from rhesus macaques were assessed via the identical method.

### 4.11 Assessment of T Cell Responses by Intracellular Cytokine Staining (ICS)

Splenocytes were resuspended and plated at a concentration of 1 × 10^7^ cells/mL. The peptide pool was introduced at a final concentration of 1 µg/mL, and the cells were co‐incubated for an additional 2 h. Subsequently, brefeldin A was added at a concentration of 5 µg/mL, and the cells were incubated overnight to inhibit cytokine secretion. Following this incubation period, the cells were harvested and subjected to viability staining utilizing Zombie NIR dye (Biolegend) to differentiate between live and dead cells. Following the addition of 5 µg/mL CD16/CD32 antibodies to inhibit nonspecific binding of Fc receptors, the mixture was incubated at 4°C for 10 min. Subsequently, fluorescent anti‐mouse CD4 and CD8 antibodies (Biolegend) were added, and the mixture was incubated at 4°C for an additional 30 min. Then, the fixation and permeabilization solution were added and incubated at room temperature for 20 min After washed with permeabilization wash buffer, APC‐tagged anti‐mouse IFN‐γ, PE‐tagged anti‐mouse IL‐2, and BV650‐tagged anti‐mouse TNF‐α antibodies were added and incubated with the cells. Over 20,000 events corresponding to CD4^+^ or CD8^+^ T cells were subsequently analyzed utilizing a CytoFLEX flow cytometer in conjunction with FlowJo software. The specific T‐cell‐mediated immune responses within peripheral blood mononuclear cell (PBMC) samples obtained from rhesus monkeys were detected via the identical experimental approach. The final concentration of peptide pool was 2 µg/mL.

### 4.12 Measurement of Antigen‐Specific AIM+ CD4 T Cells and Tfh Cells

Splenocytes were resuspended at a concentration of 2 × 10^7^ cells/mL. Then, 100 µL of the cell suspension was added into a 96‐well plate and stimulated with peptide pool (5 µg/mL/peptide) for 20 h. Afterwards, cells were stained with Zombie Violet tm Fixable Viability Kit for 15 min and then incubated with antibody cocktails for 30 min at ambient temperature. The cells were washed twice by FACS buffer and analyzed by Flow cytometry.

### 4.13 Analysis of GC B cell Response

Frequencies of class‐switched (IgD‐IgM‐) gE‐specific GC B cells were assessed by flow cytometry. Cells from mouse spleen or lymph node were first stained by Zombie Violet Fixable Viability Kit for 15 min and washed by FACS buffer. Then, the cells were incubated with gE‐protein for 30 min at ambient temperature. After washing, cells were incubated with antibody cocktails for 30 min at ambient temperature and analyzed by flow cytometry.

### 4.14 Statistical Analysis

All the experiments were conducted independently and the specific sample sizes (n) can be found in the figure legends. Data were presented as mean ± SEM unless otherwise stated. The data were subjected to one‐way analysis of variance (ANOVA), followed by Dunnett's multiple comparisons test, utilizing the Shingrix group as the control. Statistical analyses were performed using GraphPad Prism version 10.5.0. A significance level of *p* < 0.05 was established, with the following designations for significance: * *p* < 0.05; ** *p* < 0.01; *** *p* < 0.001; ****p < 0.0001.

For the statistical analysis of long‐term immunogenicity in the rhesus macaque model, R version 4.4.1 was used. First, the tidyverse package was employed to calculate the geometric mean titer (GMT) and its 95% confidence interval (CI), or the arithmetic mean (AM) and its standard error (SEM), at each time point. Subsequent statistical inference was performed by constructing a linear mixed‐effects model (LMM) using the lme4 package, with each individual included as a random effect to analyze temporal changes in repeated‐measures data. Next, the emmeans package was utilized to compute estimated marginal means (EMMs) and conduct multiple comparisons with Tukey adjustment. Statistical significance results were generated with compact letter display (CLD) labels using the multcompView package. Finally, immunological persistence was evaluated by fitting a linear regression to the log10‐transformed titer data using the lm function, thereby calculating the half‐life of antibody titer decay.

## Funding

Self‐funded by Beijing Youcare Kechuang Pharmaceutical Technology Co., Ltd.

## Conflicts of Interest

G.S.S., K.D., Y.F.L, and Y.T.Z. have a granted patent (CN118240844B) that discloses the preparation method and application of an mRNA vaccine for VZV prevention. G.S.S. holds granted patents (CN114044741B in China, US12171876B2 in US, JP7606722B1 in Japan) pertaining to Lipid YK‐009, which delineate the characteristics of the compound, the formulations that incorporate it, and its potential applications. The remaining authors declare no competing interests.

## Ethics Approval Statement

All the animal experiments were conducted in compliance with the guidelines for the care and use of laboratory animals, and all experiments involving laboratory animals were approved by the Institutional Animal Care and Use Committees (IACUCs) of JOINN Laboratories (Beijing: No. B‐ACU24‐0281; Suzhou: S‐ACU24‐0764) and Wuxi Apptec (No. PD13‐CD059‐2024v1.0).

## Supporting information




**Supporting File**: advs73569‐sup‐0001‐SuppMat.pdf.

## Data Availability

The data that support the findings of this study are available from the corresponding author upon reasonable request.
