# Supplementary material for: Potent and Long‐Lasting Immunogenicity Generated by LNP‐mRNA gE Antigen Against Varicella Zoster Virus via an AI‐Assisted Pipeline
Source: Adv Sci (Weinh). 2025 Dec 25;13(14):e10913. doi: 10.1002/advs.202510913 (PMC12970245; doi:10.1002/advs.202510913)
Supplement: Supplementary file 1 — Supporting File: advs73569‐sup‐0001‐SuppMat.pdf. [file ADVS-13-e10913-s001.pdf]

Supporting Information

Potent and long-lasting immunogenicity generated by LNP-mRNA gE antigen against varicella-zoster virus via an AI-assisted pipeline

*Kai Dong[#], Fang Liu[#], Dawei Wang, Yanfen Li, Man Zhang, Yuting Zhou and Gengshen Song**

Affiliations: Beijing Youcare Kechuang Pharmaceutical Technology Co., Ltd., Beijing 100176, China;

[#]Authors contributed equally *Corresponding author

Correspondence: songgengshen@youcareyk.com

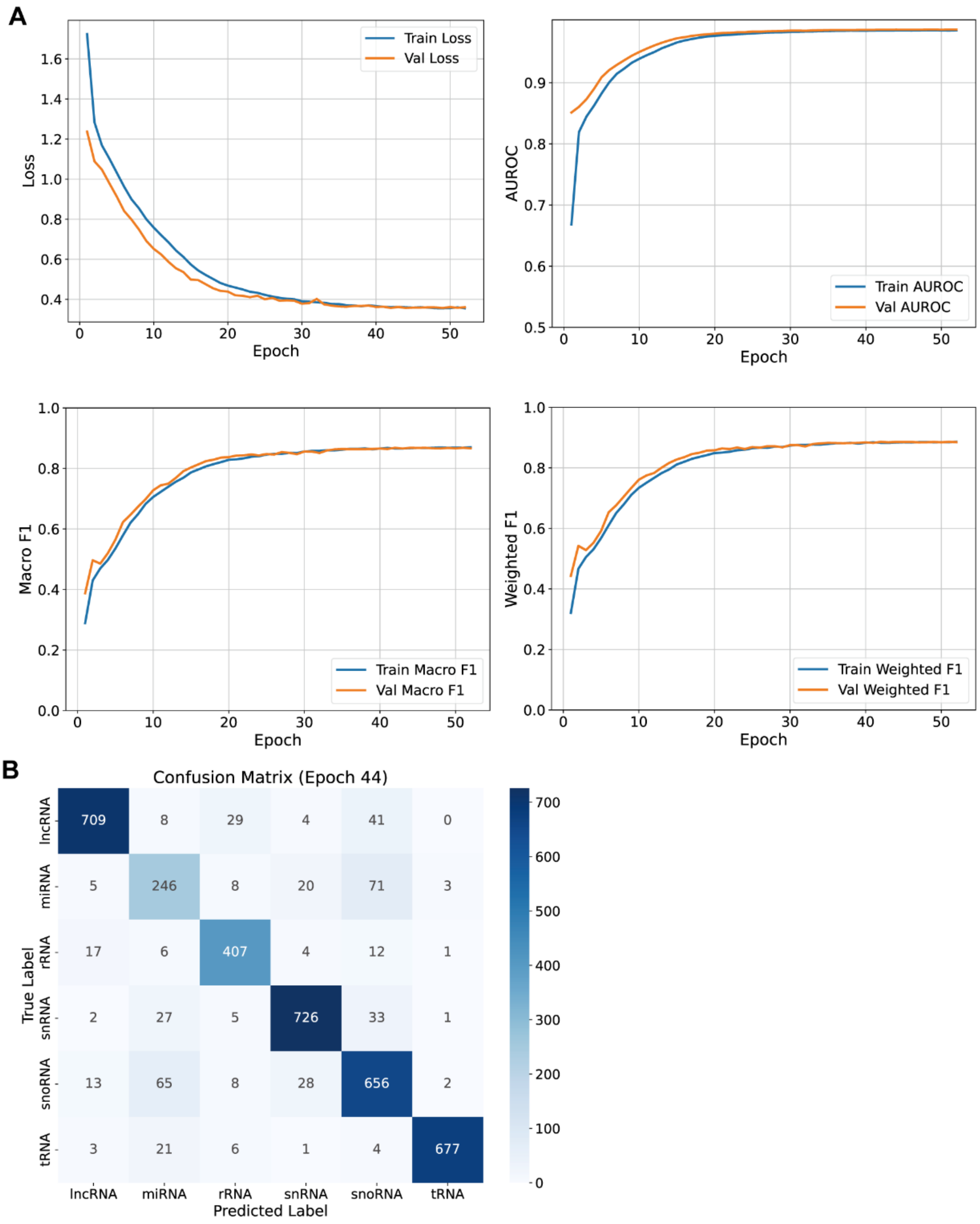

Figure S1. Learning curves (A) and confusion matrix (B) of the optimal pre-trained model.

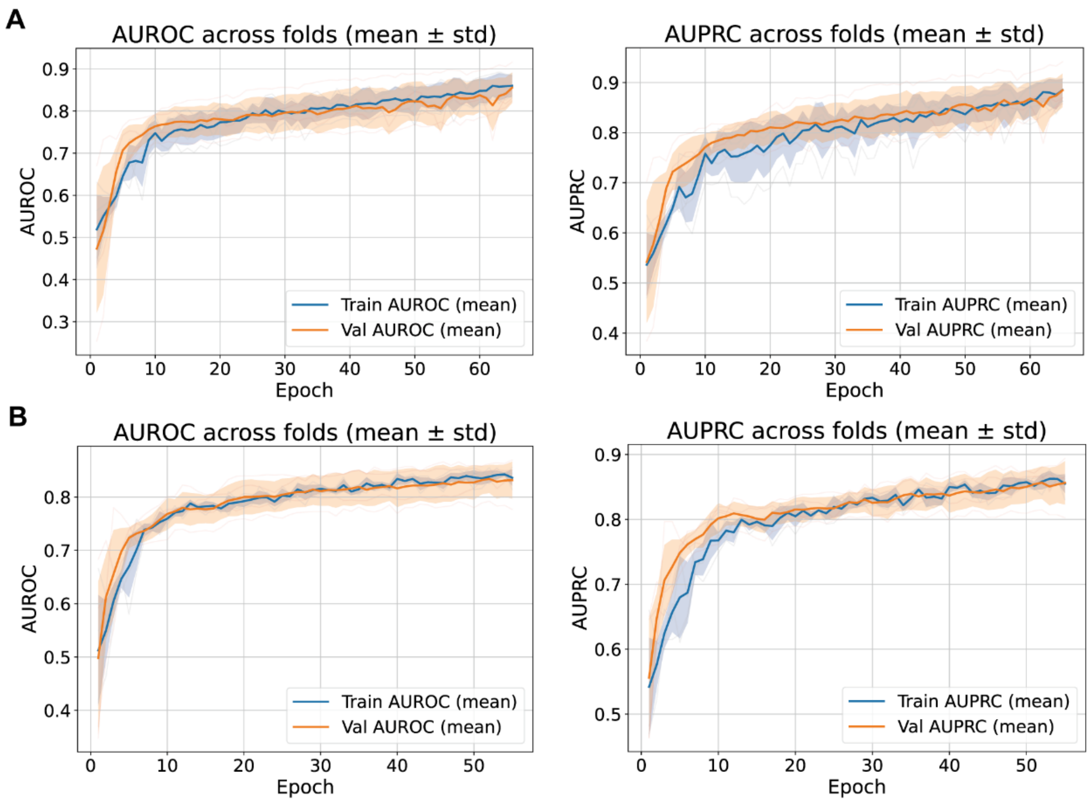

Figure S2. Cross-validation learning curves of the fine-tuned protein expression prediction model (A) and the IgG level prediction model (B).

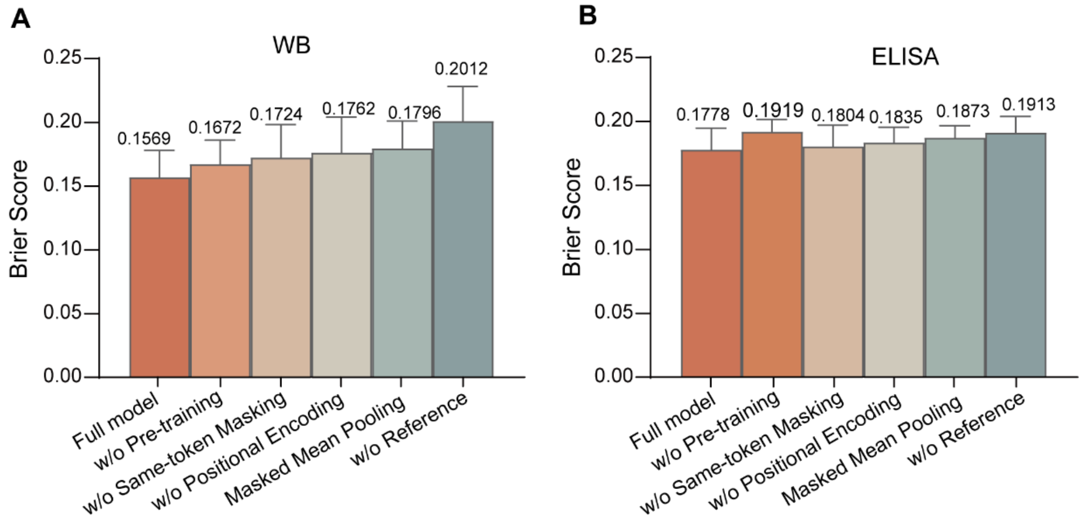

Figure S3. Mean calibration performance of the ablated model variants for the protein expression prediction model (A) and the IgG level prediction model (B) across cross-validation folds. The numbers above the columns represent the mean value of each group. Data are represented as mean ± SD.

Supplementary Figures

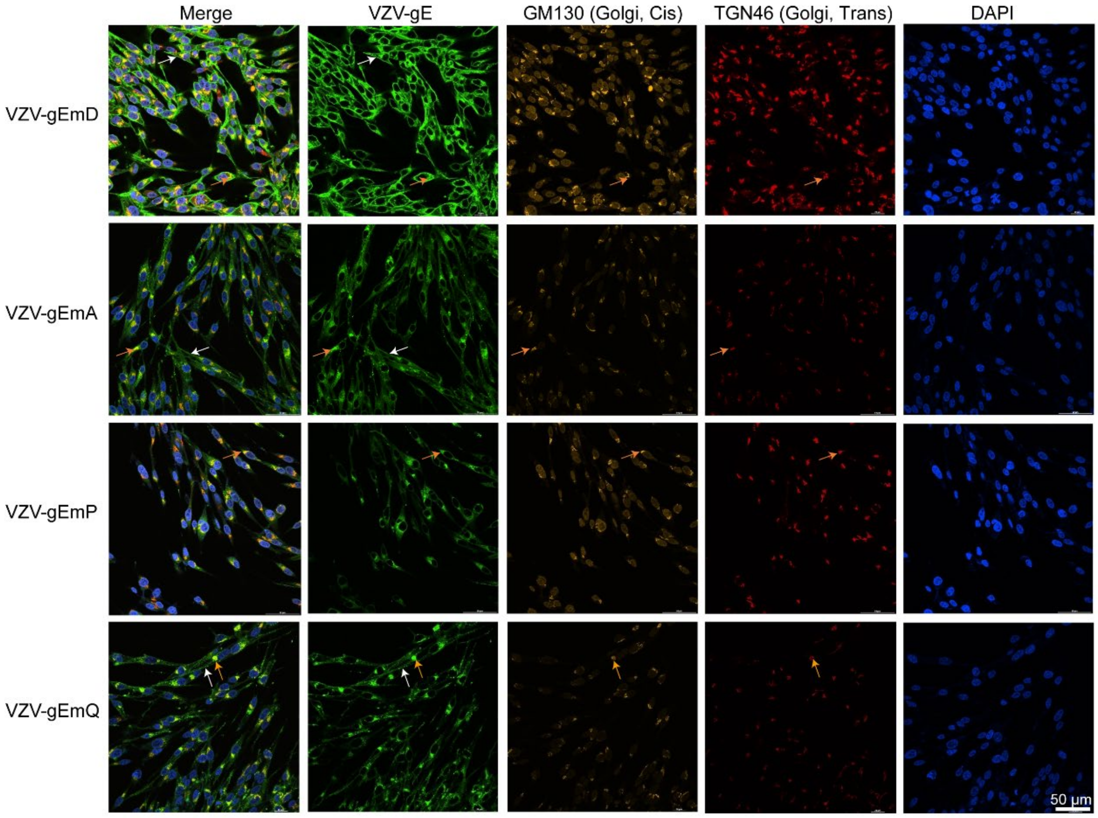

Figure S4. Global image of cellular localization and expression of antigen candidates in MeWo cells in Figure 4. gE antigen: green; *Cis*-Golgi: orange; *Trans*-Golgi: red; DAPI: blue. The white arrows point to the cell membrane. The orange arrows indicate the location of the Golgi. Scale bar: 50 µm.

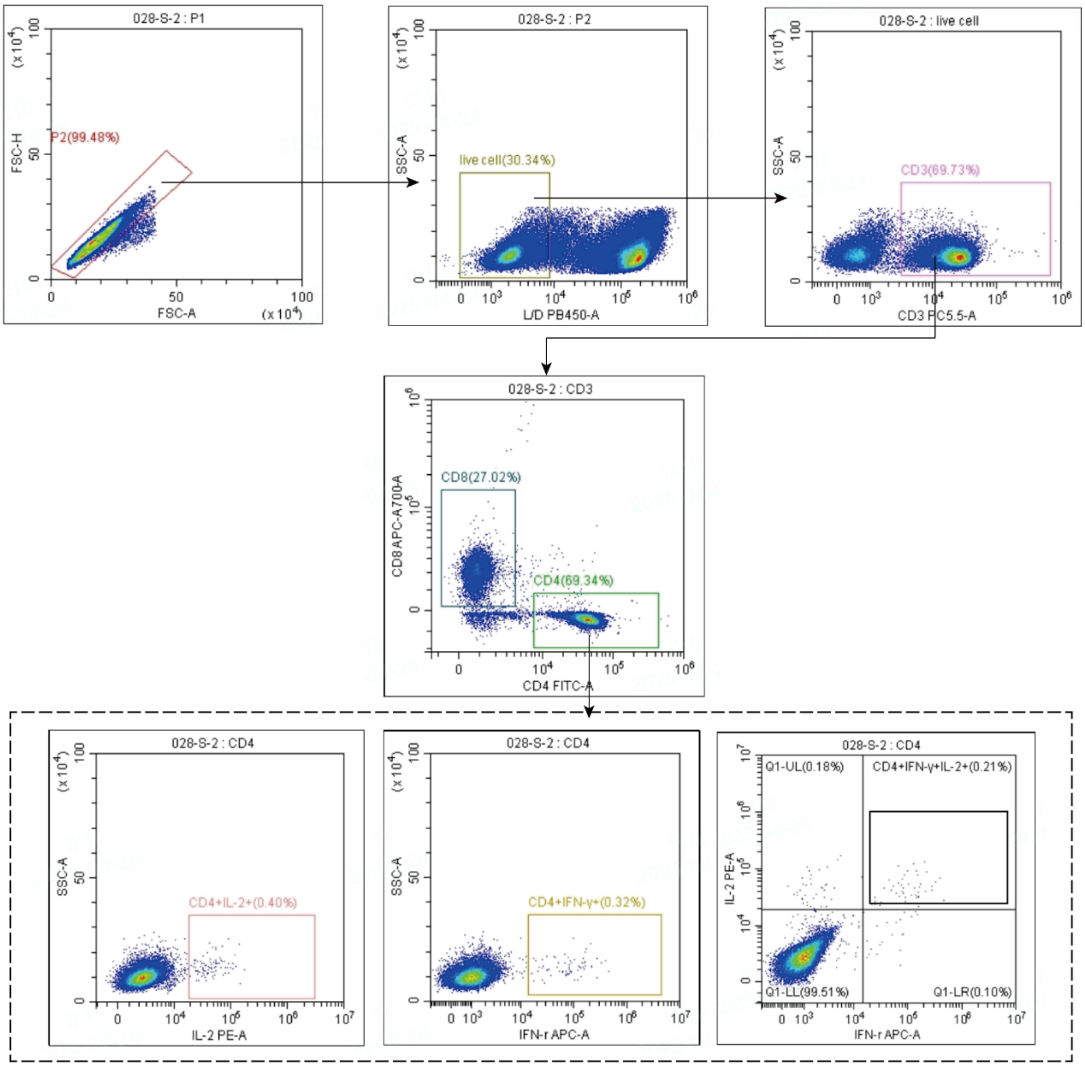

Figure S5. Representative gating strategy for flow cytometry analysis of UTR screening experiments (Figure 5E-G).

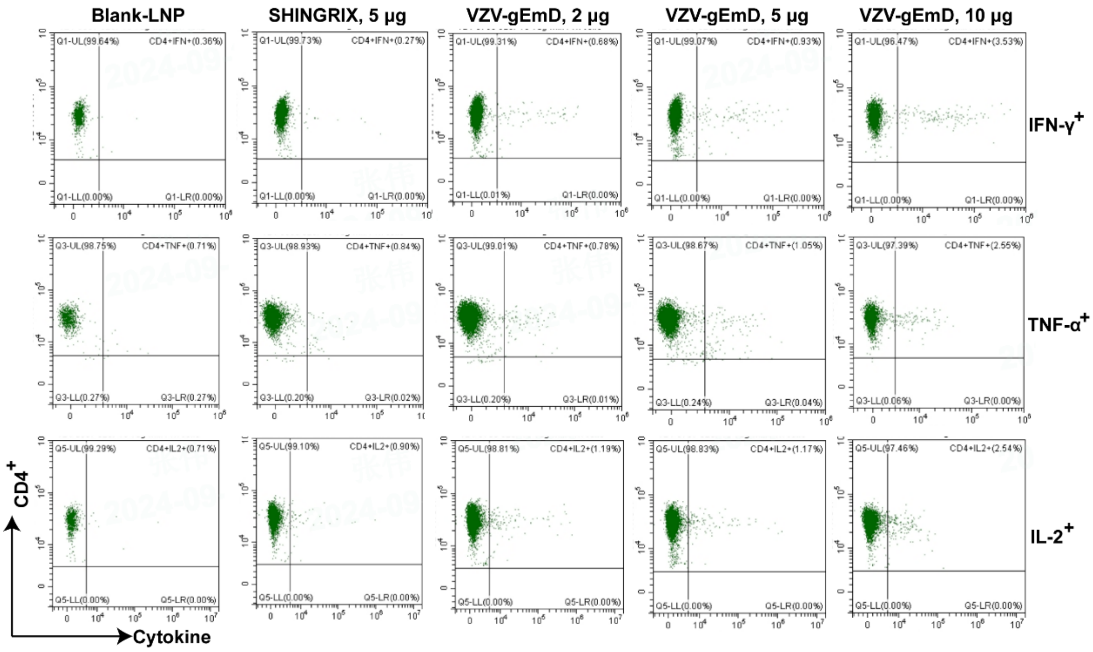

Figure S6. Flow cytometry assay for gE-specific IFN-γ-, TNF-α- and IL-2-producing $CD4^+$ T cells in BALB/c mice.

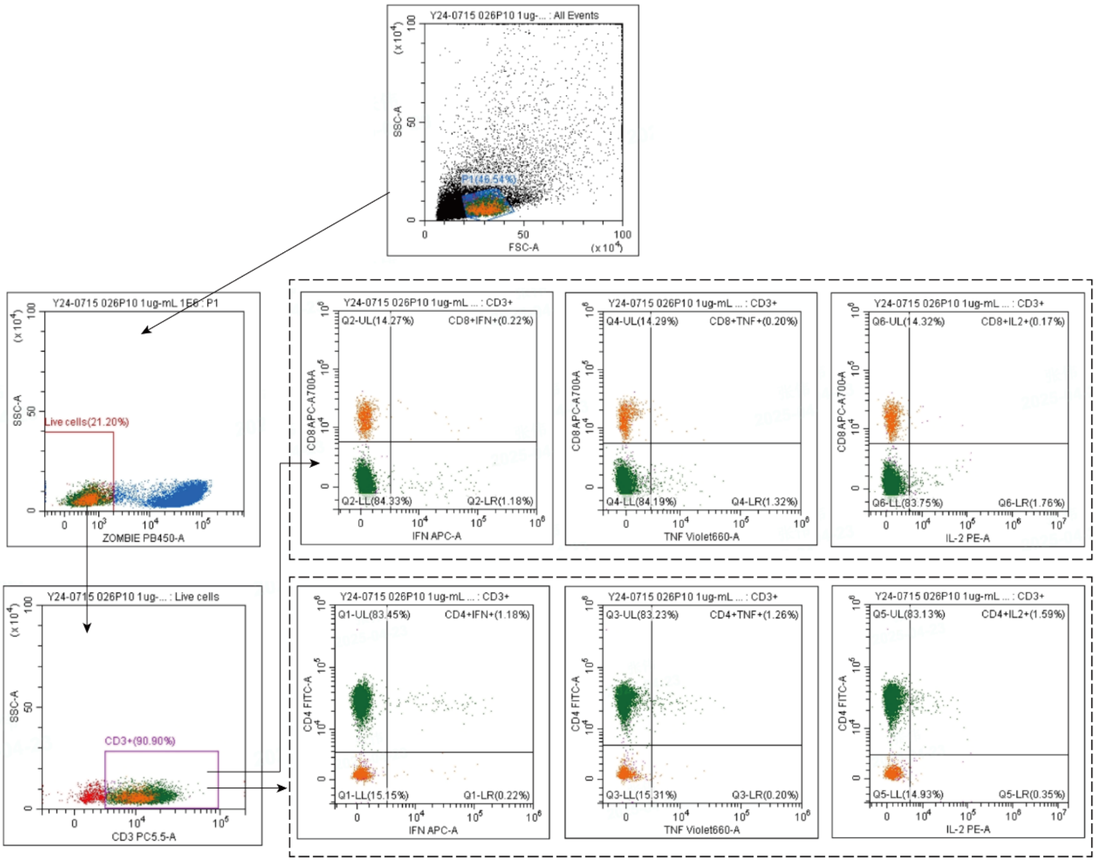

Figure S7. Representative gating strategy for flow cytometry analysis of T cell response in BALB/c mice (Figure 7G, Figure S6).

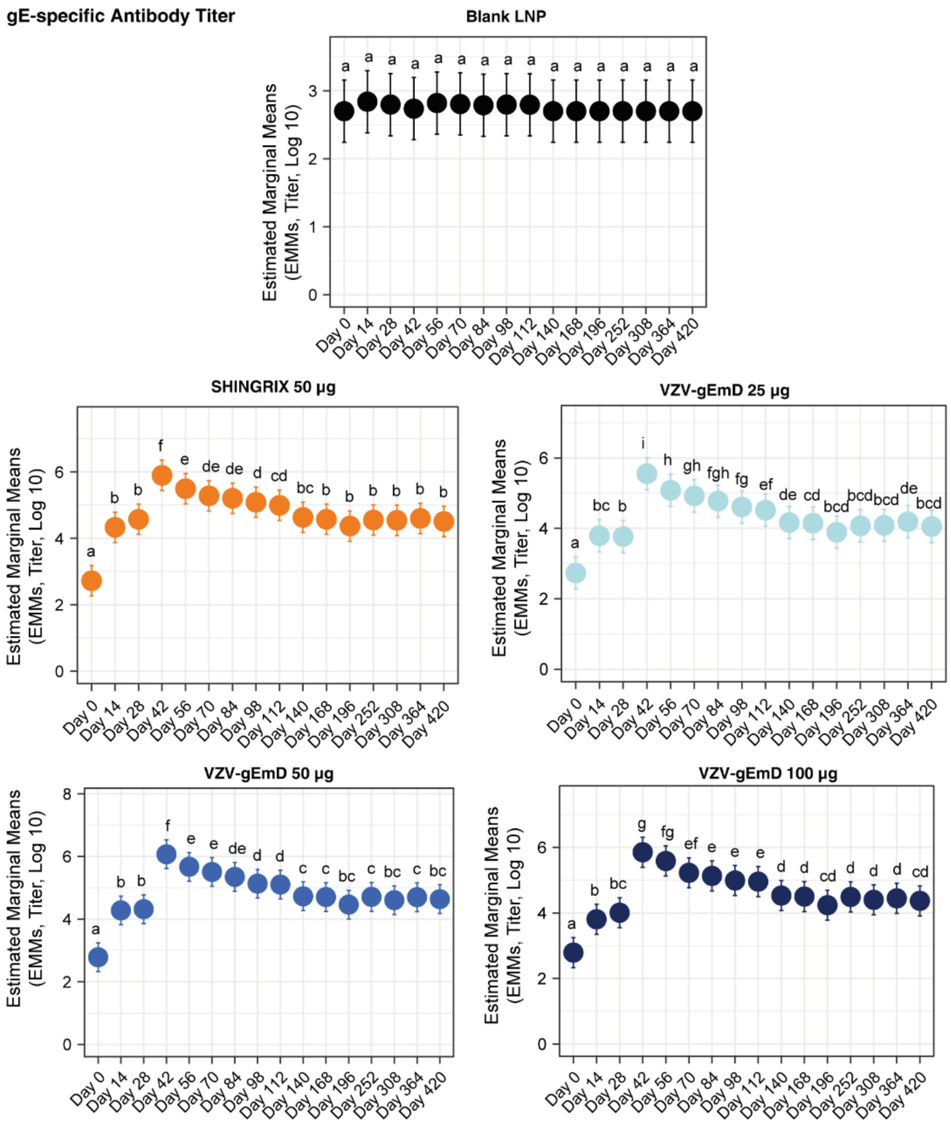

Figure S8. Longitudinal gE-specific antibody responses across vaccine groups.

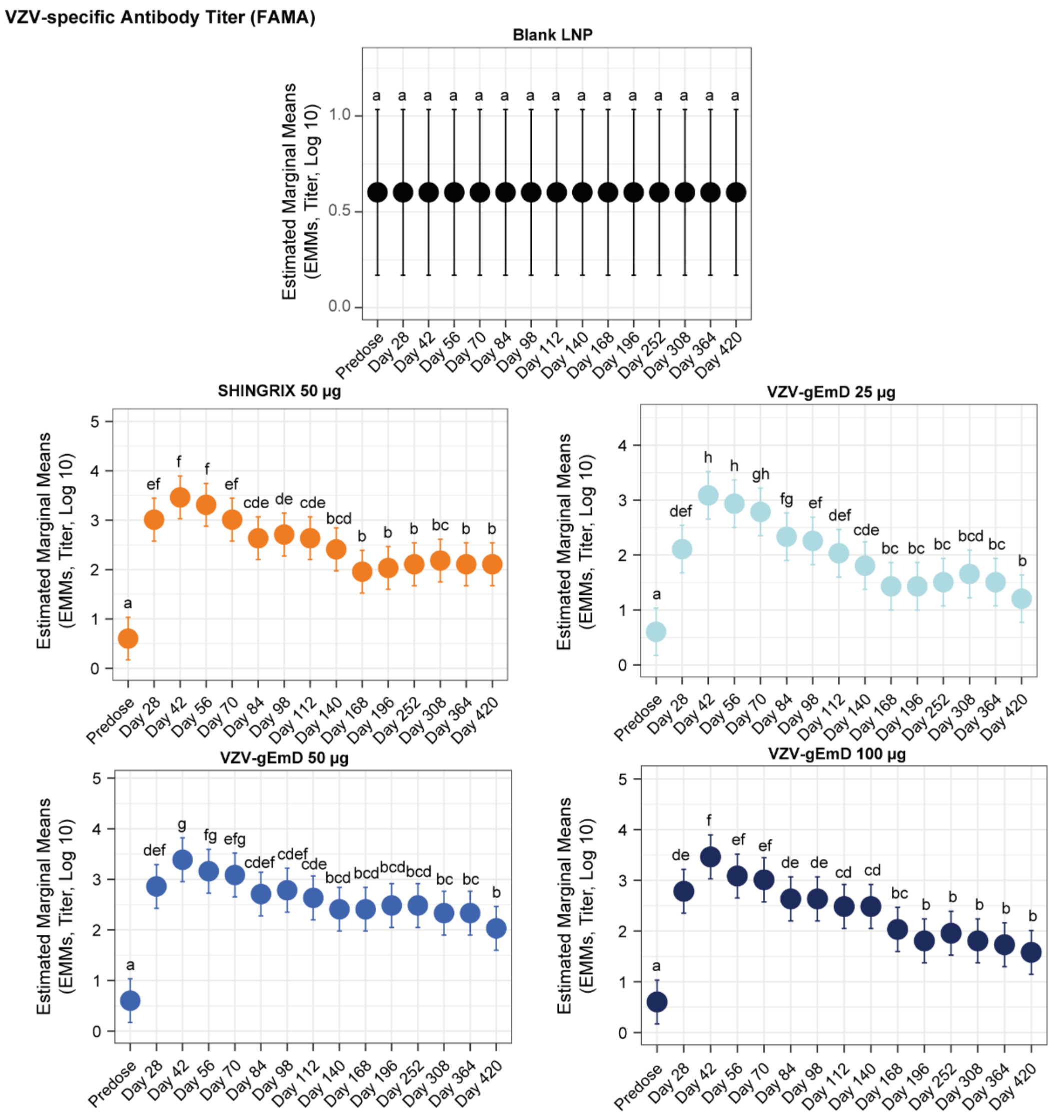

Figure S9. Longitudinal VZV-specific antibody responses across vaccine groups.

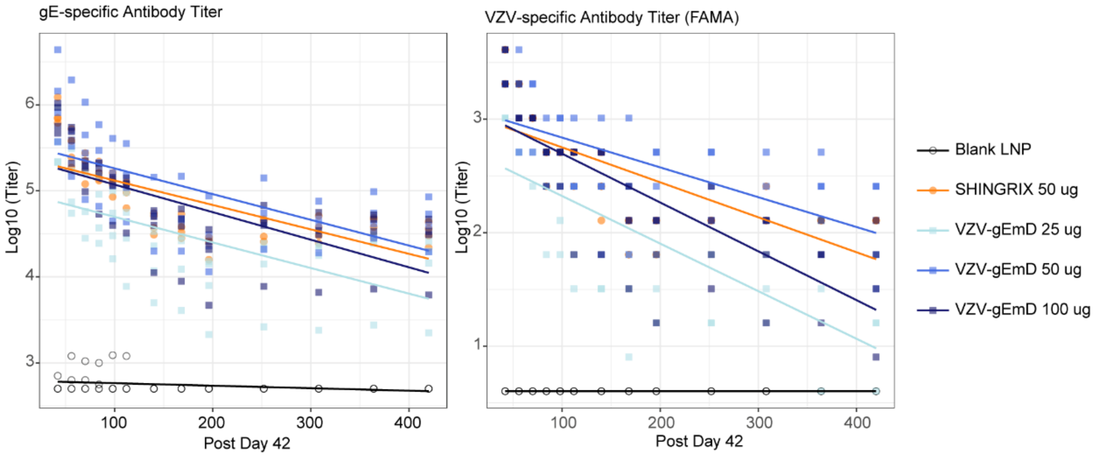

	gE-specific Antibody Titer		VZV-specific Antibody Titer (FAMA)	
	half life (days)	*p* value	half life (days)	*p* value
SHINGRIX 50 ug	105.96	7.38E-10	97.68	3.99E-10
VZV-gEmD 25 ug	101.22	3.70E-06	71.75	2.98E-09
VZV-gEmD 50 ug	100.66	9.65E-07	114.56	1.47E-07
VZV-gEmD 100 ug	93.96	4.95E-08	69.96	3.57E-12

Figure S10. Half-life analysis for gE- and VZV-specific antibody responses.

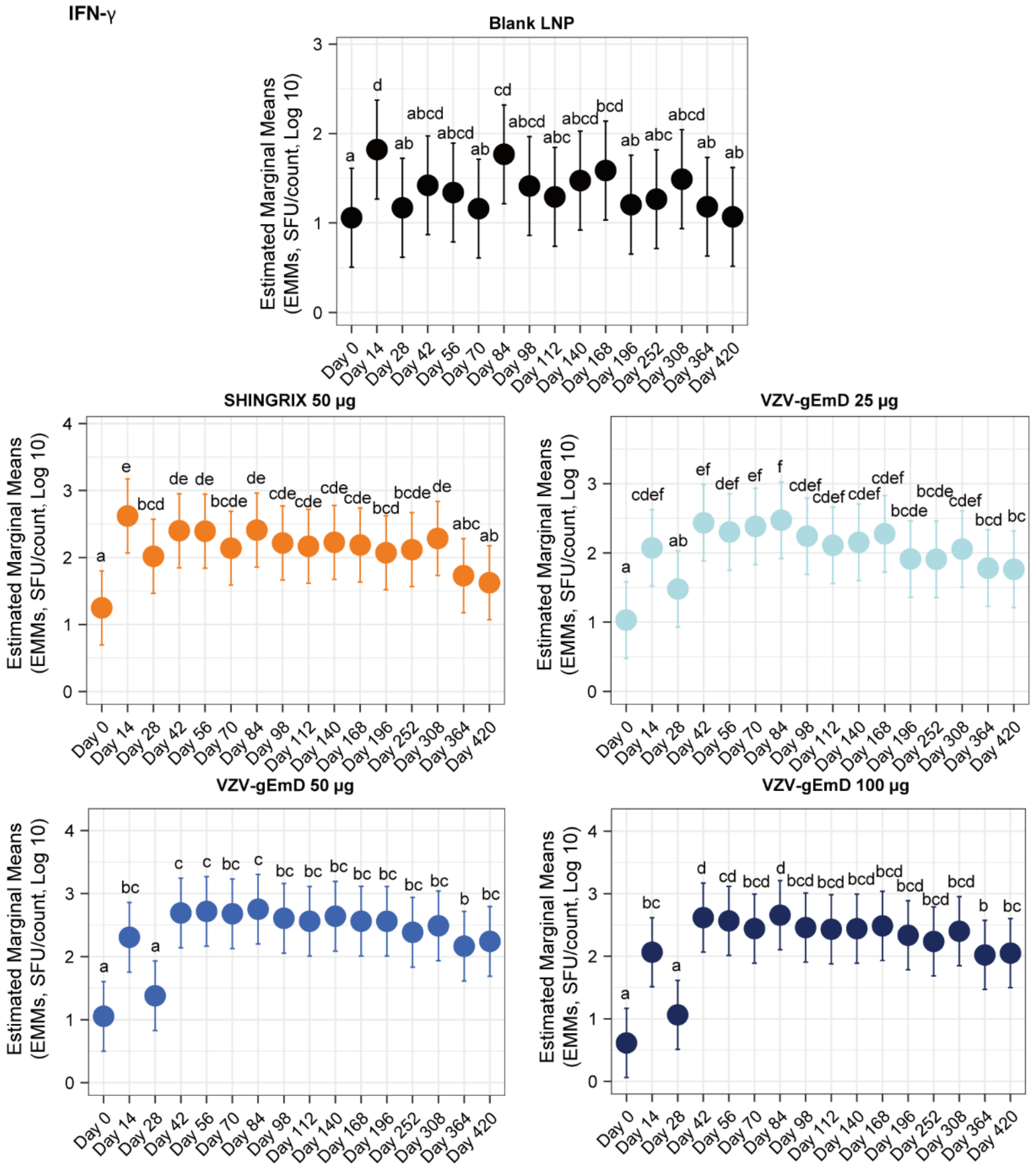

Figure S11. Longitudinal IFN-γ responses across vaccine groups.

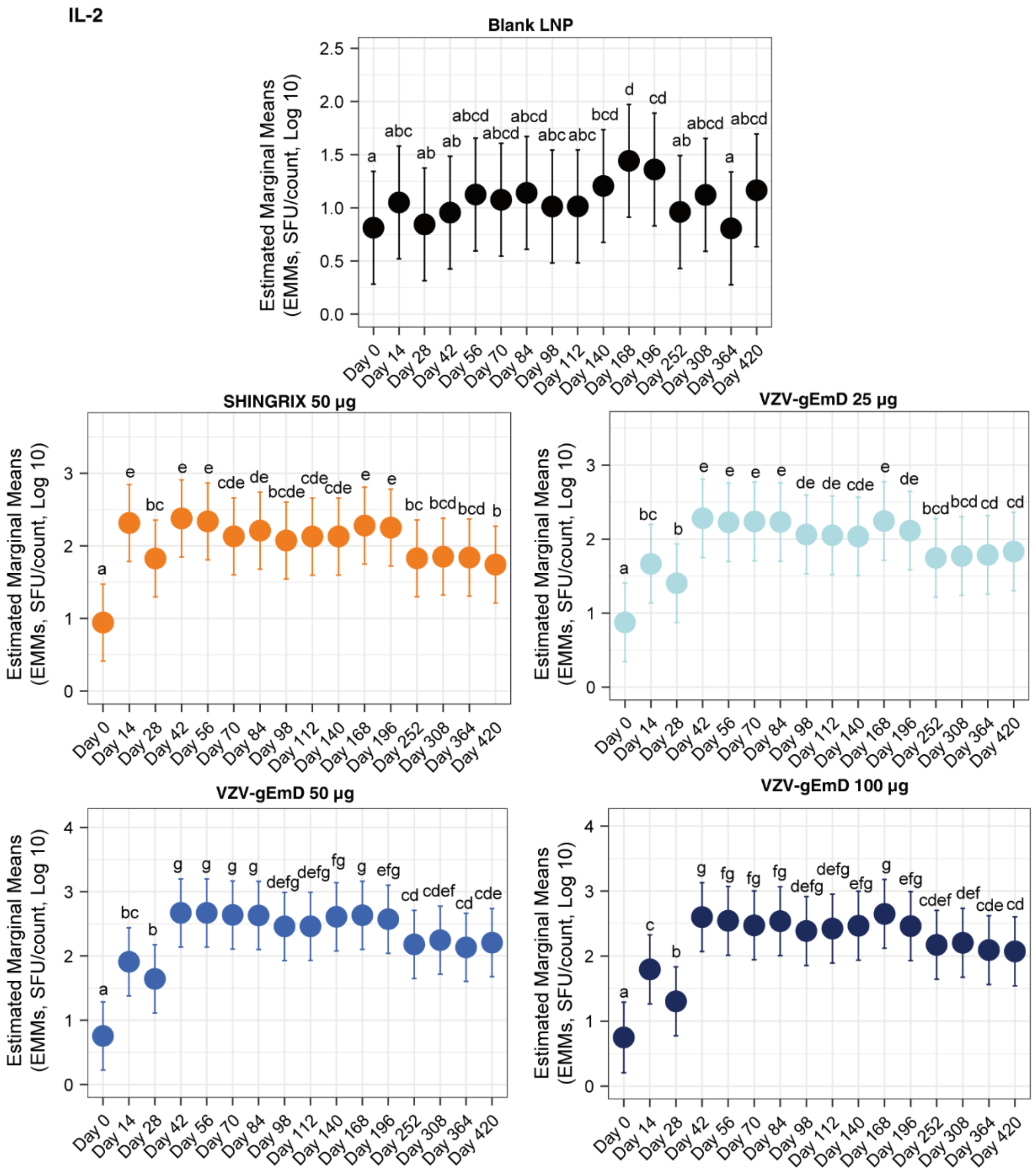

Figure S12. Longitudinal IL-2 responses across vaccine groups.

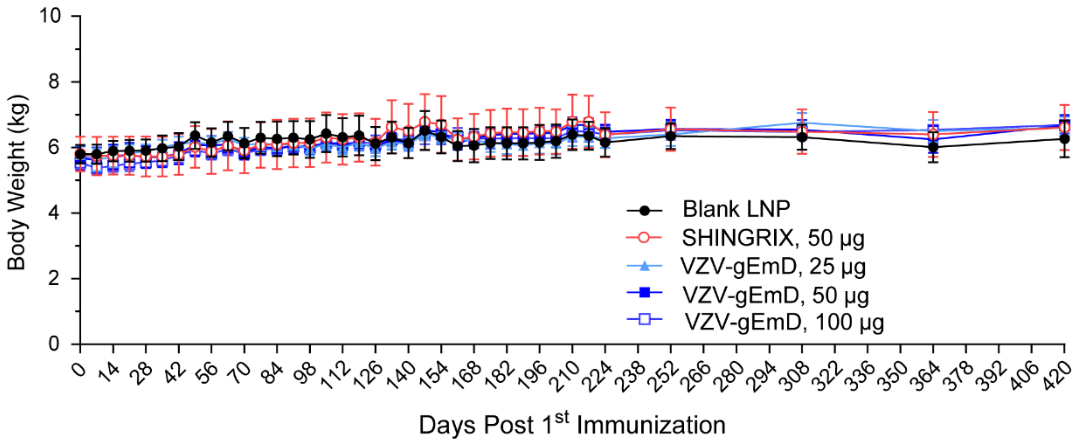

Figure S13. Body weight monitoring in rhesus macaques during the immunization period.

Supplementary Tables

Table S1. Cross-validation performance of fine-tuning tasks

Task	Protein Expression Prediction			IgG Level Prediction		
Metric	AUROC	AUPRC	Brier Score	AUROC	AUPRC	Brier Score
Fold1	0.850	0.857	0.157	0.792	0.815	0.199
Fold2	0.834	0.872	0.167	0.833	0.867	0.190
Fold3	0.916	0.942	0.123	0.869	0.894	0.159
Fold4	0.825	0.869	0.180	0.854	0.866	0.164
Mean ±SD	0.856 ±0.036	0.885 ±0.033	0.157 ±0.021	0.837 ±0.029	0.861 ±0.029	0.178 ±0.017

Table S2. Ablation study results for the protein expression prediction model on pooled out-of-fold predictions

Ablated Component	AUROC	AUCPR	Brier Score
Full Model	0.851 (0.814, 0.883)	0.880 (0.849, 0.913)	0.157 (0.137, 0.178)
w/o Pre-training	0.837 (0.798, 0.870)	0.865 (0.832, 0.898)	0.167 (0.148, 0.186)
w/o Reference	0.760 (0.714, 0.803)	0.773 (0.715, 0.826)	0.201 (0.184, 0.221)
w/o Same-token Masking	0.819 (0.778, 0.856)	0.848 (0.812, 0.884)	0.172 (0.153, 0.193)
w/o Positional Encoding	0.814 (0.775, 0.855)	0.827 (0.786, 0.870)	0.176 (0.156, 0.198)
w/o Masked Mean Pooling	0.813 (0.775, 0.851)	0.844 (0.807, 0.883)	0.180 (0.165, 0.195)

Note: Values in parentheses "()" represent the 95% confidence interval (95% CI).

Table S3. Ablation study results for the IgG level prediction model on pooled out-of-fold predictions

Ablated Component	AUROC	AUCPR	Brier Score
Full Model	0.826 (0.788, 0.863)	0.856 (0.815, 0.895)	0.178 (0.157, 0.201)
w/o Pre-training	0.796 (0.751, 0.837)	0.822 (0.776, 0.867)	0.192 (0.170, 0.214)

w/o Reference	0.774<br>(0.728, 0.816)	0.775<br>(0.716, 0.839)	0.191<br>(0.170, 0.213)
w/o Same-token Masking	0.812<br>(0.769, 0.851)	0.841<br>(0.800, 0.882)	0.180<br>(0.161, 0.203)
w/o Positional Encoding	0.804<br>(0.758, 0.847)	0.825<br>(0.779, 0.872)	0.184<br>(0.163, 0.205)
w/o Masked Mean Pooling	0.796<br>(0.749, 0.837)	0.824<br>(0.778, 0.870)	0.187<br>(0.165, 0.210)

Note: Values in parentheses “()” represent the 95% confidence interval (95% CI).

Table S4. Characterization of VZV-gE mRNA drug substance

Sample Name	VZV-gE mRNA drug substance			
	A260/A280	mRNA Integrity (%)	mRNA Sequence Length (nt)	5’ Capping Efficiency (%)
VZV-gEmA	1.90	80.3	1978	97.4
VZV-gEmB	1.91	89.8	2138	96.4
VZV-gEmD	1.88	86.5	2137	95.2
VZV-gEmH	1.90	83.5	2112	98.5
VZV-gEmK	1.90	86.0	2144	95.7
VZV-gEmL	1.90	83.2	2104	96.4
VZV-gEmN	1.92	86.7	1991	96.3
VZV-gEmP	1.92	86.2	2000	96.6
VZV-gEmQ	1.90	88.6	2030	95.0
VZV-gEmD UTR0	1.90	89.4	2140	99.3
VZV-gEmD UTR1	1.88	91.2	2141	98.2
VZV-gEmD UTR2	1.90	89.1	2173	99.6
VZV-gEmD UTR3	1.89	86.3	2099	98.5
VZV-gEmD UTR4	1.90	89.3	2114	98.9

Table S5. Characterization of VZV-gE mRNA-LNP for *in vivo* studies

Sample Name	VZV-gE mRNA-LNP							
	mRNA Integrity (%)	mRNA Sequence Length (nt)	Encapsulation Efficiency (%)	Nanoparticle Size (%)	Polydispersity Index (PDI)	ζ-potential (mV)	Ethanol residue	Bacterial endotoxin (EU/ml)
VZV-gEmA	78.5	2035	95.0	74	0.19	-8	<LOQ	<50
VZV-gEmB	87.6	2161	95.6	63	0.10	-6	<LOQ	<50
VZV-gEmD	85.8	2103	95.5	66	0.09	-5	<LOQ	<50
VZV-gEmH	83.0	2168	94.7	71	0.20	-5	<LOQ	<50
VZV-gEmK	86.1	2121	95.3	57	0.09	-6	<LOQ	<50
VZV-gEmL	82.3	2161	94.6	72	0.19	-8	<LOQ	<50
VZV-gEmN	85.8	1977	96.2	65	0.09	-7	<LOQ	<50
VZV-gEmP	82.1	2023	96.1	64	0.08	-5	<LOQ	<50
VZV-gEmQ	88.7	2000	95.1	58	0.10	-5	<LOQ	<50
VZV-gEmD UTR0	87.3	2170	95.0	56	0.09	-8	<LOQ	<50
VZV-gEmD UTR1	88.3	2163	95.8	58	0.08	-6	<LOQ	<50
VZV-gEmD UTR2	86.4	2210	96.1	60	0.11	-5	<LOQ	<50
VZV-gEmD UTR3	83.8	2123	96.2	58	0.09	-6	<LOQ	<50
VZV-gEmD UTR4	87.2	2121	90.2	76	0.09	-6	<LOQ	<50

Note: Ethanol residue detection method: Gas Chromatography (GC), Limit of Quantitation (LOQ): 0.02%; Bacterial endotoxin detection method: Kinetic Chromogenic Assay.

Table S6. Statistical analysis results in Figure 3C and D

VZV-gEmD vs.	IL-2		IFN-γ	
	Significance	*p*-value	Significance	*p*-value
VZV-gEmB	****	<0.0001	**	0.0056
VZV-gEmH	n.s.	0.9554	n.s.	0.1209
VZV-gEmK	***	0.0002	n.s.	0.1899
VZV-gEmL	n.s.	0.1391	n.s.	0.4656
VZV-gEmA	****	<0.0001	**	0.0025
VZV-gEmN	***	0.0001	n.s.	0.0578
VZV-gEmP	n.s.	0.9554	n.s.	0.2144
VZV-gEmQ	****	<0.0001	****	<0.0001

Table S7. Minimum free energy (MFE) predictions of UTRs

Name	MFE
UTR0	0.00kcal/mol
UTR1	-0.10 kcal/mol
UTR2	-22.30kcal/mol
UTR3	-3.30kcal/mol
UTR4	-6.20kcal/mol

Table S8. Anti-PEG antibody assessment in rhesus macaques

**Samples**	**Time Point**
	**Day42**
Blank-LNP 1	-
Blank-LNP 2	-
Blank-LNP 3	-
Blank-LNP 4	-
VZV-gEmD 25 µg 1	-
VZV-gEmD 25 µg 2	-
VZV-gEmD 25 µg 3	-
VZV-gEmD 25 µg 4	-
VZV-gEmD 50 µg 1	-
VZV-gEmD 50 µg 2	-
VZV-gEmD 50 µg 3	-
VZV-gEmD 50 µg 4	-
VZV-gEmD 100 µg 1	-
VZV-gEmD 100 µg 2	-
VZV-gEmD 100 µg 3	-
VZV-gEmD 100 µg 4	-
Note：1. - → Negative Results in Screening Assay.	

Table S9. Microscopic pathological changes associated with the VZV-gEmD and blank-LNP at the termination of the Dosing Phase (Day 32)

Site	Gender		Male				Female			
	Group		1	2	3	4	1	2	3	4
	Dose (μg /animal)		0	0	25	50	0	0	25	50
	Animal counts		10	10	10	10	10	10	10	10
**Administration Site**	Inflammatory Cell Infiltration; Intramuscular/Perimuscular	Slight	7	2	3	0	7	3	5	1
		Mild	0	7	7	8	0	7	5	8
		Moderate	0	0	0	2	0	0	0	1
	Edema; Intramuscular/Perimuscular	Slight	0	0	1	3	0	2	3	3
		Mild	0	3	3	3	0	1	1	4
		Moderate	0	0	0	0	0	0	0	2
	Fibrosis; Intramuscular/Perimuscular	Slight	0	9	4	4	0	2	5	4
		Mild	0	0	0	1	0	0	0	0
	Hemorrhage; Intramuscular/Perimuscular	Slight	0	2	1	2	0	1	0	3
		Mild	0	1	0	0	0	0	0	0
	Necrosis; Intramuscular/Perimuscular	Slight	0	1	3	7	0	2	4	3
		Mild	0	1	1	0	0	0	0	4
	Neovascularization; Perimuscular	Slight	0	4	1	2	0	3	4	4
		Mild	0	1	1	1	0	0	0	0
	Degeneration/ Necrosis; Muscle Fiber	Slight	1	2	7	6	4	6	4	7

Note: Group 1 - Negative control, Group 2 - blank-LNP, Group 3-4 - VZV-gEmD.

Table S10. Microscopic pathological changes associated with the VZV-gEmD and blank-LNP at the termination of the Recovery Phase (Day 57)

**Site**	Gender		Male				Female			
	Group		1	2	3	4	1	2	3	4
	Dose (μg/animal)		0	0	25	50	0	0	25	50
	Animal counts		5	5	5	5	5	5	5	5
**Administration Site**	Inflammatory Cell Infiltration; Intramuscular/Perimuscular	Slight	1	2	0	1	0	0	1	1
	Fibrosis; Intramuscular	Slight	0	1	1	0	0	0	0	1
	Degeneration/Necrosis; Muscle Fiber	Slight	1	0	0	1	0	0	0	0

Note: Group 1 - Negative control, Group 2 - blank-LNP, Group 3-4 - VZV-gEmD.

Table S11. Architecture hyperparameters of the RDTransformer

	Hyperparameters	Value
Token Embedding	Vocabulary Size	65
	Embedding Dimension	64
	Pre-training Initialization	Normal distribution (mean=0, std=0.02)
Positional Encoding	Encoding Type	Sinusoidal
	Maximum Sequence Length	2048
Transformer Encoder	Number of Encoder Layers	2
	Number of Attention Heads	8
	Feedforward Hidden Dimension	192
	Dropout Rate	0.3 for Pre-training<br>0 for Fine-tuning
	Pre-training Initialization	Xavier uniform for weights (all linear layers), zero bias; LayerNorm defaults
Feature Pooling	Method	Global Mean Pooling for Pre-training<br>Padding Masked Max Pooling for Fine-tuning
Pre-training Classifier	Dropout Rate	0.3
	Output Dimension	6
	Initialization	Xavier uniform for weights, zero bias

Fine-tuning Classifier	Hidden Dimension	64
	Dropout Rate	0.2 for Protein Expression Prediction<br>0.1 for IgG Level Prediction
	Output Dimension	1
	Initialization	Xavier uniform for weights, zero bias; LayerNorm defaults
Pre-training Trainable Parameters	-	88,006
Fine-tuning Trainable Parameters	-	91,969

Table S12. Model training hyperparameters

Category	Parameter	Value for Pre-training	Value for Fine-tuning
Training Procedure	Optimizer	AdamW	AdamW
	Batch Size	128	8
	Learning Rate	8e-5	3e-5 for WB<br>5e-5 for ELISA
	Max Training Epochs	500	65 for WB CV<br>64 for WB Full Training<br>55 for ELISA CV<br>52 for ELISA Full Training
	Early Stopping Patience	10	N/A
	Early Stopping Delta	1e-3	N/A
Learning Rate Schedule	Linear Warm-up Start Factor	0.1	0.01
	Linear Warm-up End Factor	1.0	1.0
	Linear Warm-up Epochs	7	5
	Cosine Cycle Epochs	50	60 for WB<br>50 for ELISA
	Minimal Learning Rate	3e-6	5e-5 for WB<br>3e-5 for ELISA

Regularization	Weight Decay (L2)	1e-3	1e-3
Gradient Clipping	Gradient Clipping Norm	1.0	1.0
Loss Function	-	Weighted Categorical Cross-Entropy	Binary Cross-Entropy

Note: WB and ELISA denote the prediction models for protein expression and IgG levels, respectively.
